# Comparative changes in sugars and lipids show evidence of a critical node for regeneration in safflower seeds during aging

**DOI:** 10.3389/fpls.2022.1020478

**Published:** 2022-10-27

**Authors:** Lanyu Zhou, Lijie Lu, Chao Chen, Tao Zhou, Qinghua Wu, Feiyan Wen, Jiang Chen, Hugh W. Pritchard, Cheng Peng, Jin Pei, Jie Yan

**Affiliations:** ^1^ State Key Laboratory of Southwestern Chinese Medicine Resources, Chengdu University of Traditional Chinese Medicine, Chengdu, China; ^2^ College of Pharmacy, Chengdu University of Traditional Chinese Medicine, Chengdu, China; ^3^ Department of Comparative Plant and Fungal Biology, Royal Botanic Gardens, Kew, Wakehurst, Ardingly, United Kingdom; ^4^ Chinese Academy of Sciences, Kunming Institute of Botany, Kunming Yunnan, China

**Keywords:** safflower seeds, critical node, regeneration, sugar, lipid, controlled deterioration treatment (CDT)

## Abstract

During seed aging, there is a critical node (CN) where the population viability drops sharply. Exploring the specific locations of the CN in different species of plants is crucial for understanding the biological storage properties of seeds and refining seed life span management. Safflower, a bulk oil crop that relies on seeds for propagation, has a short seed life. However, at present, its biological characteristics during storage are not clear, especially the changes in metabolic capability and cell structures. Such knowledge is needed to improve the management of safflower seed life span and effective preservation in gene banks. Here, the seed survival curve of oilseed safflower under the controlled deterioration conditions of 60% relative humidity and 50°C was detected. The seed population showed an inverted S shape for the fall in germination. In the first 12 days of aging, germination remained above 86%. Prior to the CN at approximately day 10 (C10), when viability was in the “plateau” interval, seed vigor reduced at the same imbibition time point. Further analysis of the changes in sugar concentration found that the sucrose content decreased slowly with aging and the content of raffinose and two monosaccharides decreased abruptly at C10. Differentially metabolized lipids, namely lysophospholipids [lyso-phosphatidylcholine (LPC) and lyso-phosphatidylethanolamines (LPE)] and PMeOH, increased at day 3 of aging (C3). Fatty acid content increased by C6, and the content of phospholipids [phosphatidylcholines (PC), phosphatidylethanolamines (PE), and phosphatidylinositols (PI) and glycolipids [digalactosyl diacylglycerol, monogalactosyl diacylglycerol, and sulphoquinovosyl diglycerides (SQDG)] decreased significantly from C10. In addition, the activities of raffinose hydrolase alpha-galactosidase and the glyoxylate key enzyme isocitrate lyase decreased with seed aging. Confocal microscopy and transmission electron microscopy revealed shrinkage of the seed plasma membrane at C10 and the later fragmentation. Seedling phenotypic indicators and 2,3,5-triphenyltetrazolium chloride activity assays also verified that there were significant changes in seeds quality at the CN. In summary, the time point C10 is a CN during seed population aging. Before the CN, sugar and lipid metabolism, especially fatty acid metabolism into sugar, can make up for the energy consumed by aging. After this point, the seeds were irreversibly damaged, and their viability was greatly and rapidly reduced as the cell structure became increasingly destroyed.

## Introduction

Safflower (*Carthamus tinctorius* L.), a multipurpose plant, is well known as an excellent oil seed crop (Zhang et al., 2016). Currently, safflower is widely planted in more than 60 countries and regions on all continents except Antarctica (Mani et al., 2020; Li et al., 2021). Originally, safflower was cultivated to extract dyes (Knowles, 1980), and later, it became an important source of edible oil due to the seeds being rich in unsaturated fatty acids (FAs; oleic acid and linoleic acid) (Dajue and Mündel, 1996; Amini et al., 2014). The seed oil content of safflower averages 40% of dry weight (DW), which is comparable with that of sunflower, olive, and peanut (Kumar et al., 2016). Linoleic acid is one of the most valuable FAs in edible oils and accounts for more than 70% of the total FAs in safflower seeds (Siddiqi et al., 2011). Safflower seed oil has excellent medicinal and nutritional values but brings a high risk for seed storage as oil seeds rich in unsaturated FAs tend to be sensitive to peroxidation (Pukacka and Kuiper, 1988; Goel and Sheoran, 2003; Shuai et al., 2017). Species characteristics (genotype and phenotype) and storage conditions determine seed longevity and the relative rate of aging (Bailly, 2004; Amini et al., 2014). For the safflower seeds, the germination rate sharply declined, and the viability was completely lost after 14 months of storage under ambient conditions (Kumari, 2009). Safflower seed also has relatively short longevity in the soil seed bank, in which no viable seeds were found after 2 years (McPherson et al., 2009). Loss of seed viability under natural conditions is a major challenge to agricultural production and productivity (Gao et al., 2016).

The complex and changing environment creates instability in production, which also increases the need for efficient and effective seed preservation (Argosubekti, 2020). Global warming is one of the major concerns of the world, with different models predicting its detrimental effects on crops (Ahmad et al., 2021a; Tan and Shibasaki, 2003). For example, climate change is projected to reduce the global production of four major crops (wheat, maize, soybean, and rice) in current agricultural regions (Rosenzweig et al., 2014; Ostberg et al., 2018). Moreover, climate simulations for West Africa (2000–2009) showed an increase in temperature of approximately 1°C and a reduction in regional average yields of 10%–20% for millet and 5%–15% for sorghum in both crop models (Sultan et al., 2019). Oilseed crops are also seriously threatened by high-temperature stress, especially seed yield and quality (Ahmad et al., 2021c). Thus, seed yields of camelina and canola dropped by 39% and 38%, respectively, under high temperatures (Jumrani and Bhatia, 2018; Ahmad et al., 2021b). Although safflower shows drought tolerance, deficit irrigation during the vegetative growth period seriously affected the yield and quality of safflower seed, compared with full irrigation (Hussain et al., 2016). Stress under dry-hot conditions at maturity has been reported to limit safflower production by diminishing photosynthesis and crop nitrogen uptake (Koutroubas and Papakosta, 2010). As an annual plant, part of the harvested safflower seeds is stored and the other part is used for later sowing (Singh and Nimbkar, 2006), which means that it is necessary to effectively preserve safflower germplasm resources. Plant conservation and sustainable utilization are critical to safeguard food and nutritional security now and in the future, in the face of adverse environments and rapidly fluctuating meteorological conditions (Pilling et al., 2020). Genebanks play a key role in this system of protection and use (Singh et al., 2012).

Germplasm preservation in gene banks requires monitoring of seed viability, especially as the regeneration thresholds are approached. Seed deterioration in storage is unavoidable, especially for oil crops (Hong et al., 2005; Probert et al., 2009; Ballesteros et al., 2019). Seed aging is a complex biological trait, such that the deterioration of seeds during storage can be separated into four events: reduction of protective capacity against oxidative stress, damage to the plasma membrane, depletion of reserves, and destruction of genetic material (Ebone et al., 2019). To research the longevity, storage characteristics, deterioration, etc., of seeds (Hu et al., 2012; Colville et al., 2012), accelerated aging tests (Probert et al., 2009; Davies et al., 2016) are commonly used. In the controlled deterioration treatment (CDT), seeds are exposed to an aging environment of controlled relative humidity (RH) and temperature (Sew et al., 2016). By setting the humidity to 60% RH, with unsaturated LiCl solution, and temperature treatment to 45°C or 60°C, it is possible to generate a single seed survival curve that can be used to compare species’ responses and may be, sometimes, a surrogate for seed longevity during dry storage in seed banks (Newton et al., 2014).

Often a critical node (CN) in the seed survival curve is observable where the rate of decreasing viability and seedling vigor shifts from a slower to more rapid rate. For most seed crops, accessions are regenerated when seed viability falls below 85% of the initial germination in active collections, as determined by vigor monitoring (Karrfalt, 2008; Matthews et al., 2012). Studies have shown that the time point in the survival curve for rice seeds at which germination approaches 82% can be used as a CN (Shenzao et al., 2018). In two wheat seedlots [storage-tolerant (ST) genotype and storage-sensitive (SS) genotype], the CN was found to be 82 ± 8% and 81 ± 8% germination, respectively (Chen et al., 2021). ST seeds exhibited a longer “plateau” phase than SS seeds, indicating that ST seeds were more resistant to aging. Therefore, seed populations with different aging tolerances reach the CN at different times. A CN is approximately 85% germination but is not fixed at that level. Recent research has focused on proteomic and mitochondrial changes in seeds around a CN (Yin et al., 2017; Chen et al., 2019). For example, at CNs of regeneration, seed respiration is restricted, mitochondria are damaged, and there is oxidative damage, which are manifested as marked changes in protein abundance and protein carbonylation levels (Yin et al., 2016). However, the basic state of metabolism at the CN is still unclear. As the main energy storage of oil crops, lipids and sugars are directly related to the germination of seeds. During the imbibition of oilseeds, the hydrolysis of triacylglycerols releases glycerol and FAs and converts the latter into sugars, which then support seed germination (Theodoulou and Eastmond, 2012). The low ability of storage lipids to metabolize into sugars during the germination of aged soybean seeds leads to the failure of normal germination and seedling formation (Zhou et al., 2019). In addition, the oxidation and hydrolysis of internal lipids in aged wheat seeds have been linked to the death of long-term aged seeds (Wiebach et al., 2020). Finally, lipid oxidation is often associated with cell membrane damage, which is also thought to be a major event in seed aging (Ratajczak et al., 2019), e.g., through changes in membrane permeability (Ratajczak et al., 2015).

To explore this question, we propose a hypothesis based on previous research: substance metabolism and changes in plasma membrane structure play decisive roles in the different stages of seed aging. We determined the seed survival curve of safflower seeds using CDT and observed the aging process in relation to energy reserve consumption and plasma membrane damage. The quality of safflower seeds showed quite different properties before and after the “plateau” stage of the aging curve, as revealed by soluble sugar content determinations using high-pressure liquid chromatography (HPLC), enzyme activities measured by a Micro Assay Kit, and lipidomics by electrospray ionization mass spectrometry (ESI-MS/MS) (Li et al., 2008; Lin et al., 2021). Seed cell membranes were also observed using the laser confocal microscopy and transmission electron microscopy (TEM). Our results provide biological evidence for a CN during safflower seed population deterioration and help to define safe storage periods for the regeneration of safflower seeds.

## Material and methods

### Seed collections

Seeds of safflower (*Carthamus tinctorius* L.) were collected from Jianyang of Sichuan Province, China, in June 2020. The collected seeds were dried under natural conditions. The original germination level was 98%, and the moisture content (MC) was 0.077 g H_2_O g^−1^ DW. Before the experiments, the seeds were held under medium-term storage conditions of <45% RH and 4°C in sealed containers.

### Experimental design and plant management

A one-factor completely randomized design was used for the CDT, with seeds placed in a sealed box with 60% RH and 50°C temperature. The different levels are the time spent in the aging environment. More than 500 seeds were randomly taken at regular intervals to serve as a level. The pot experiment was a completely random design with 19 pots randomly placed in a greenhouse environment. The experimental pot was 48 cm long, 18 cm wide, and 15 cm high with 2/3 volume peat soil. For each treatment, 75 seeds were evenly sown in peat soil in pots, covered with a thin layer of soil after sowing, and watered well. Soil moisture was observed every 2 days after sowing and the soil kept moist as necessary. The greenhouse provided 75% RH, a day/night temperature of 20°C/15°C, and photoperiod of 8 h day/16 h night during the plant growth period.

### Plant sampling

In the CDT aging experiment, seed samples were taken on days 0, 1, 2, 3, 4, 5, 6, 7, 8, 9, 10, 11, 12, 13, 16, 20, 22, 25, 28, 34, 37, and 40 of treatment (and coded as C0–C13, C16, C20, C22, C25, C28, C34, and C40). Sufficient intact seeds were stored briefly at 4°C for use in the germination tests, assessment of seedling formation, and the tetrazolium uptake assay. Considering that there are great differences between the chemical composition of the safflower seed coat and cotyledon, the coat was removed before the sugar content determination, lipidomics, and enzymatic activity tests. A certain amount of all treated samples was stored at −80°C after the seed coat was removed and frozen in liquid nitrogen prior to chemical analysis. A part of the more intact seed kernel with the seed coat removed was fixed in FAA solution (38% formaldehyde/glacial acetic acid/70% alcohol, 5:5:90, v/v) for laser confocal electron microscopy observation, and a part was fixed in 3% glutaraldehyde solution for TEM and kept at 4°C. In the pot experiment, 30 seedlings with true leaves were taken for each (aging) level, with 10 seedlings per replicate, after 15 days of seedling growth.

### Controlled deterioration treatment

The seeds were surface-disinfected by immersing in 5% sodium hypochlorite solution (CAS: 7681-52-9) for 5 min and then rinsed three times with distilled water. After that, seeds were rapidly air-dried until they reach the original MC. A LiCl solution was made by adding 300 g of anhydrous lithium chloride (CAS: 7447-41-8) to 1 L of distilled water to maintain an environment RH of 60% in the sealed plastic boxes (Bewley et al., 2012). The seeds were equilibrated above the LiCl solution in sealed boxes at 20°C for 24 h, and this defined day 0 (C0) and the control for the aging treatment. Thereafter, the sealed boxes and seeds were transferred to 50°C, and the viability loss was judged by germination tests. Finally, samples with different aging days were obtained and subjected to a range of physiological, chemical, and microscopic assessments.

### Seed germination

Before proceeding to germination, seeds were disinfected with 5% sodium hypochlorite solution as described above. Seeds were germinated separately as four replicates of 50 seeds per sample. Seeds were sown on 1% agar in petri plates at 25°C and under dark conditions. Germination was recorded when the radicles emerged through the seed coat by more than 2 mm. Germination rate was calculated at different imbibition times (imbibed for 18, 20, 24, 28, 32, 36, 40, 44, 48, 52, 56, 72, and 120 h), and the final statistical time of the germination rate was the seventh day of germination.

### Determination of sugars

The sugars were extracted according to a published protocol (Zhou et al., 2022). Samples were taken at different aging time points; the seed embryos were freeze-dried (FDU-2110, EYELA, Japan), then ground in a clean mortar, and put into an Eppendorf tube. A 200-mg aliquot of the sample was put into a 10-ml graduated centrifuge tube with the addition of 5 ml of 80% ethanol and placed in an 80°C water bath for 10 min, shaken three to five times in the middle of the process. Next, the supernatant was collected after centrifugation at 5,000g for 5 min. The above operation was repeated and the two supernatants were combined. The extracts were filtered through 0.22-μm water phase filters (Jinteng, China). Sugars were measured directly in the extract by HPLC using a Waters binary HPLC system (Waters 1525-2707, Milford, USA) equipped with a refractive index detector (2414, Waters, Milford, USA). Analytical conditions were as follows: column Agilent Hi-Plea Ca (8% cross-linked), 300 × 7.7 mm, 8 µm in diameter (Agilent Technologies, Inc., USA); column temperature, 85 °C; mobile phase, Milli-Q water; and flow rate, 0.6 ml min^−1^. Data were collected and processed by the Waters chromatography station DataApex. Sugars were identified by comparison with retention times and coelution of authentic standard solutions.

### Comparative lipidomics

Three replicates per treatment level (C0, C6, C10, C16, C22, C28, and C34) were assessed for whole seeds that had been shelled. About 15 mg of lyophilized powder was taken of each sample, and 80 μl (10 μg/ml) of an internal standard was added. In addition, 2 ml dichloromethane and 2 ml methanol were added, and the mixture was whirled for 1 h. The mixture was left overnight to precipitate the protein. Then, 2 ml of dichloromethane and 1.6 ml of ultra-pure water were added, and the mixture was subjected to vortex centrifugation. The lower clarified liquid was taken off, and the remaining upper phase was extracted by adding 4 ml of dichloromethane and repeated twice. The lower liquid fractions were combined, blown-dried with nitrogen, redissolved with 1 ml of isopropanol, and passed over a 0.22-μm organic filter membrane prior to testing.

Analyses were performed using a ultra performance liquid chromatography (UPLC) system (Thermo Fisher, Germany) coupled with a triple quadrupole tandem mass spectrometer (Triple TOF, AB Sciex, USA). All samples were quantitatively analyzed in positive and negative modes using high-resolution LC-MS and were structurally analyzed in the positive mode using LC-MS/MS.

### Measurement of enzyme activity

The activity of alpha-galactosidase (α-GAL) and isocitrate lyase (ICL) was determined with kits from Solarbio (α-GAL, Beijing, China; ICL, Beijing, China). α-GAL decomposes p-nitrobenzene-α-D-galactopyranoside to generate p-nitrophenol, which has a maximum absorption peak at 400 nm. The activity of α-GAL was calculated by measuring the rate of increase in absorbance. About 0.1 g of tissue in dry seeds (C0, C6, C10, and C16) was weighed, 1 ml of extract was added, and the mixture was homogenized in an ice bath. After 15,000g centrifugation at 4°C for 20 min, the supernatant was taken and placed on ice for testing. The extracted enzyme solution and the substrate p-nitrophenol-α-D-galactopyranosyl solution were quickly mixed and reacted accurately in a water bath at 37°C for 30 min. Sodium carbonate solution was added and mixed thoroughly to terminate the enzyme reaction. After standing for 2 min, the absorbance was measured at 400 nm. The p-nitrophenol solution (5 μmol/ml) was diluted with distilled water with a standard solution of 800, 200, 100, 50, 25, 6.25, and 0 nmol/ml for testing.

ICL catalyzes the degradation of isocitrate into glyoxylic acid and succinic acid. Glyoxylic acid and nicotinamide adenine dinucleotide (NADH) generate ethanol and nicotinamide adenine dinucleotide (NAD) under the action of lactate dehydrogenase (LDH). NADH has a characteristic absorption peak at 340 nm. Monitoring the decreased rate of absorbance at 340 nm can indirectly reflect the activity of ICL. After the seeds were imbibed for 10 h, the seed coat was peeled off and quickly frozen with liquid nitrogen. The sample treatment was similar to the above operation. The substrate and the sample are accurately reacted in a water bath at 25°C for 2 min, and the absorbance value changes before and after the reaction were recorded at 340 nm. The consumption of 1 nmol NADH per gram of tissue in the reaction system per minute was defined as one unit of enzyme activity.

### Laser confocal microscopy and transmission electron microscopy

FM4-64 was purchased from Thermo Fisher Scientific (CA, USA). An aliquot of 1 μl of FM4-64 stock solution [5 mM, dimethyl sulfoxide (DMSO)] was added to 1 ml of water to prepare 5 μM working fluid. The freehand-cut safflower embryo slices (C0, C6, C10, C16, C22, and C34) were incubated in FM4-64 working solution for 30 min and then rinsed several times with deionized water. Thereafter, the slices were placed under a ZEISS LSM 700 confocal laser scanning microscope for fluorescence detection and analysis. FM4-64 excitation light is 555 nm, emission light is 630 nm, and the fluorescence parameters should be consistent between different treatments. Ten biological replicates were performed for each seed aging germination level.

After prefixing with a 3% glutaraldehyde, the tissue (C0, C6, C10, and C16) was postfixed in 1% osmium tetroxide, dehydrated in series acetone, infiltrated in Epox 812 for a longer time, and embedded. The semi-thin sections were stained with methylene blue, and ultrathin sections were cut with diamond knife and stained with uranyl acetate and lead citrate. Sections were examined with a JEM-1400-FLASH transmission electron microscope.

### Evaluation of seed vigor and seedling establishment

Seeds after different aging treatments (C0, C10, C11, C16, and C22) were collected and imbibed for 6 h at 25°C in the dark. After dry blotting the surface of the seeds dried with absorbent paper, seeds were gently divided in half along the hilum. Seeds were incubated in a 1% (w/v) aqueous solution of 2,3,5-triphenyltetrazolium chloride (CAS: 298-96-4) in the dark at 35°C for 1 h. At the same time, the control group (C0) was treated, and the staining was checked and photographed.

The root system of seedlings (C0–C13, C16, C20, C22, C25, and C28) was scanned with a root system scanner (Zhongjing ScanMaker i800plus, China), and statistics were made. The belowground parts were placed in an oven at 105°C for 30 min, then placed in an oven at 80°C to constant weight, and weighed with an analytical balance. Subsequently, the below biomass, root length, and root surface area were quantified.

### Statistical analysis

The data including germination levels, fresh weight, and lipid and sugar quantification results were analyzed using one-way ANOVA and Student’s t-test (SPSS 19.0). Lipid characterization and quantification were performed using software such as MSDIAL version 4.00, PeakView 2.1, and MultiQuant 3.0.2. Germination level and sugar content curves were fitted using origin2021 (OriginPro Learning Edition). ImageJ software was used to measure the length of radicles.

## Results

### Germination of aging safflower seeds

**Figure 1:**
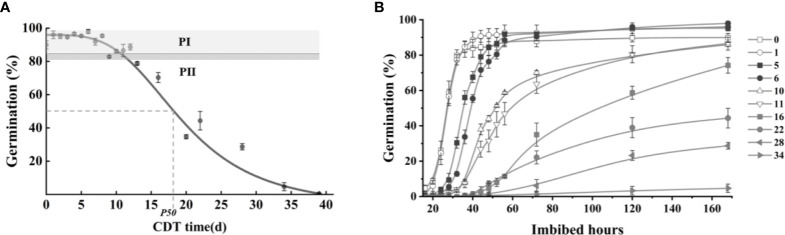
The various germination levels of aged safflower seeds. **(A)** Germination level of the seeds in different aging days: 0–13, 16, 20, 22, 28, 34, 37, and 40. **(B)** Germination level of aged seeds at different imbibition times. Data are mean ± SE. CDT, controlled deterioration treatment.

Germination was measured on deteriorated safflower seeds as a function of CDT time, and seed viability (seed germination percentage) was plotted to create a seed survival curve (**Figure 1A**). The survival curve of safflower seeds presents an inverted S shape. Seed germination at the “plateau” stage of the seed survival curve, such as C0 and C6, was determined as 87% and 90%, respectively, at 72 h of imbibition. The comparable value for C10 was 58% germination, which was significantly lower than that of unaged (C0) seeds (**Figure 1B**). For seeds aged up to 8 days, germination was between 90% and 98%, whereas at C10 and C16, germination was 85% and 75%, respectively. From C16 onward, the germination level continued to fall, reaching only 4.7% for C34 seeds and 1% in C37 seeds, and seed viability was completely lost by C40.

### The concentration of soluble sugars in aged safflower seeds

**Figure 2:**
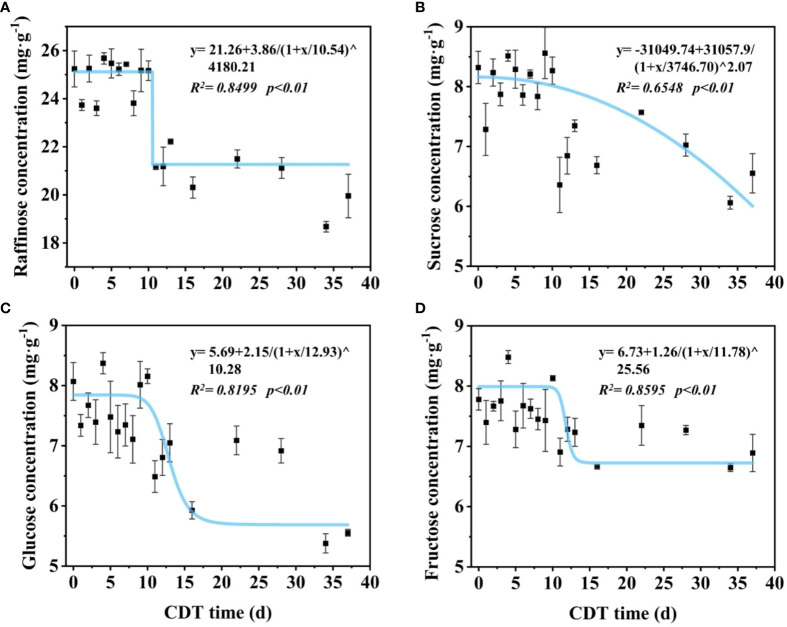
Effects of aging days on the concentration of raffinose **(A)**, sucrose **(B)**, glucose **(C)**, and fructose **(D)** in safflower seeds. The curve was simulated according to the logistic function. Seeds were sampled on aged 0–13, 16, 22, 28, 34, and 37 days. Each symbol represents the average of three replicates ± SE. CDT, controlled deterioration treatment.

We further investigated the changes of soluble sugar concentration in seeds aged for different time periods (**Figure 2**). The predominant sugar present in seeds was raffinose, and the concentration could be divided into two time stages (**Figure 2A**). For C0 to C10 seeds, raffinose was about 25 mg/g, whereas in C11 seeds and onward, the level decreased significantly to about 20 mg/g. Compared with C0 seeds, the raffinose concentration of C11 decreased significantly to 21.15 mg/g, which is a decrease of 16.2% (**Figure S2A**). Through to C11, the sucrose concentration showed a gradual decrease, from about 8.56 to 6.06 mg/g, which is a decrease of 27.2% (**Figure 2B**). There was a significant difference in the concentration between C0 and C11 seeds (**Figure S2B**). Glucose and fructose decreased as seed vigor reduced, similar to the change trend for raffinose concentration, and could be roughly divided into two time stages (**Figures 2C, D**).

### Changes in lipid species and relative content respond inconsistently to CDT

On the basis of the results of germination tests and changes in sugar concentration, representative aged seeds were selected for lipid determination using the ESI-MS/MS lipid profiling approach. We identified 182 molecular species, including three major classes: the FA classes, the glycolipid [glycerolipids: DG and TG; galactolipids: monogalactosyl diacylglycerol (MGDG), SQDG, and digalactosyl diacylglycerol (DGDG)], and the phospholipids (PC, PE, PI, PG, PA, PMeOH, Lyso PC, and Lyso PE) (**Table 1**).

**Table 1:** The total amount of lipid (nmol/g fresh weight) in each head group class in aged (C0, C3, C6, C10, C16, C22, C28, and C34) safflower seed.

Lipid	C0	C3	C6	C10	C16	C22	C28	C34
FA	1,235.84 ± 156.70^g^	1,282.00 ± 70.63^g^	1,766.02 ± 83.25^f^	2,611.20 ± 34.18^e^	3,416.10 ± 176.90^d^	4,461.41 ± 126.34^c^	5,449.08 ± 192.47^b^	5,927.79 ± 184.54^a^
LPC	32.49 ± 0.87^e^	55.57 ± 4.48^d^	70.70 ± 5.48^c^	77.63 ± 1.08^bc^	82.55 ± 0.88^ab^	92.52 ± 4.26^a^	100.23 ± 3.65^a^	91.20 ± 3.38^a^
LPE	14.70 ± 0.48^g^	22.79 ± 1.43^f^	31.17 ± 1.89^e^	37.55 ± 0.41^d^	43.65 ± 1.80^c^	51.45 ± 1.16^b^	58.99 ± 1.56^a^	56.09 ± 1.03^a^
PC-O	26.75 ± 2.14^e^	27.81 ± 0.33^de^	26.92 ± 0.80^e^	32.78 ± 1.44^cd^	36.01 ± 1.78^c^	37.47 ± 0.73^c^	45.10 ± 2.40^b^	58.62 ± 3.38^a^
PG-O	8.97 ± 0.27^cd^	9.29 ± 0.06^bc^	9.06 ± 0.36^cd^	7.92 ± 0.83^d^	10.43 ± 0.23^ab^	9.31 ± 0.44^bc^	10.46 ± 0.27^ab^	10.81 ± 0.49^a^
PMeOH	6.98 ± 0.68^c^	14.29 ± 1.52^ab^	14.77 ± 1.15^ab^	11.87 ± 1.49^b^	15.43 ± 1.90^ab^	14.75 ± 1.69^ab^	16.26 ± 0.37^a^	14.73 ± 1.82^ab^
PA	1,053.43 ± 54.88^a^	1,050.16 ± 38.61^a^	1,009.91 ± 46.34^ab^	952.06 ± 9.39^ab^	917.73 ± 42.25^bc^	806.45 ± 20.69^cd^	814.49 ± 34.63^d^	717.79 ± 17.18^d^
PC	5,550.90 ± 34.64^a^	5,256.50 ± 124.35^b^	5,130.48 ± 101.37^b^	4,773.67 ± 118.83^c^	4,407.53 ± 28.78^d^	4,020.69 ± 51.80^e^	3,864.66 ± 73.39^e^	3,215.90 ± 101.93^f^
PE	2,696.11 ± 35.54^a^	2,577.57 ± 5.52^a^	2,578.69 ± 78.17^a^	2,315.43 ± 27.56^b^	2,186.68 ± 15.43^c^	2,012.60 ± 35.38^d^	2,012.92 ± 38.91^d^	1,748.99 ± 46.44^e^
PG	70.59 ± 2.8^a^	69.25 ± 4.62^ab^	68.52 ± 2.31^ab^	67.51 ± 3.09^abc^	62.60 ± 0.62^bcd^	56.64 ± 1.99^d^	60.57 ± 1.14^cd^	57.98 ± 1.31^d^
PI	4,513.96 ± 114.29^ab^	4,435.15 ± 137.77^ab^	4,650.09 ± 47.86^a^	4,328.42 ± 24.89^b^	4,001.66 ± 29.32^c^	3,878.56 ± 82.68^c^	3,819.04 ± 48.49^cd^	3,616.63 ± 75.89^d^
DGDG	8.84 ± 0.46^a^	8.63 ± 0.46^a^	8.76 ± 0.07^a^	7.38 ± 0.43^b^	7.17 ± 0.48^b^	6.50 ± 0.63^bc^	6.65 ± 0.19^b^	5.36 ± 0.24^c^
MGDG	3.81 ± 0.24^a^	2.98 ± 0.19^b^	3.42 ± 0.35^ab^	2.21 ± 0.16^c^	2.20 ± 0.30^c^	1.83 ± 0.18^cd^	1.76 ± 0.09^cd^	1.47 ± 0.05^e^
SQDG	23.25 ± 0.82^a^	19.85 ± 1.58^b^	19.58 ± 1.04^b^	18.71 ± 1.15^bc^	15.93 ± 0.55^d^	16.17 ± 0.55^cd^	16.73 ± 0.14^cd^	10.86 ± 0.68^e^
DG	18,141.56 ± 939.01^ab^	17,295.10 ± 937.37^b^	18,051.67 ± 1,398.89^ab^	19,803.36 ± 743.97^ab^	18,860.80 ± 395.58^ab^	17,936.89 ± 1,017.47^b^	19,544.07 ± 279.34^ab^	2,0634.63 ± 839.69^a^
TG	386,062.34 ± 8,350.50^ab^	380,989.55 ± 16,452.35^b^	427,363.23 ± 16,914.55^a^	403,320.02 ± 5,083.69^ab^	426,432.85 ± 4,934.41^a^	387,361.86 ± 20,970.19^ab^	376,097.88 ± 20,057.34^b^	390,531.48 ± 15,180.92^ab^
PC: PE	2.06 ± 0.03	2.04 ± 0.04	2.00 ± 0.04	2.06 ± 0.07	2.02 ± 0.02	2.00 ± 0.04	1.92 ± 0.01	1.84 ± 0.03

Values in each lipid group marked with different letters are significantly different (p < 0.05). Values are means ± SE (n = 3).

As the heat map of lipid profiles reveals, there were substantial step-by-step differences, affected by the different artificial aging days (**Figure S1**), such that the internal lipid levels and composition of safflower seeds varied with aging days. However, neither the lipid composition, the acyl chain length, the double-bond index, nor the PC/PE ratios changed significantly. With aging, the isotype lipid of CDT safflower seeds changed slowly but consistently (**Table 1**). To sum up, the levels of most lipids increased in the first half of the aging experiment, and then, some began to decrease in the latter half of seed aging. The content of FA, PMeOH, PC-O, LPC, and LPE reveals a significant increase. In contrast, the PC, PE, PI, PA, PG, DGDG, MGDG, and SQDG contents all showed an obvious decrease. There was no significant difference in the content of DG and TG.

**Figure 3:**
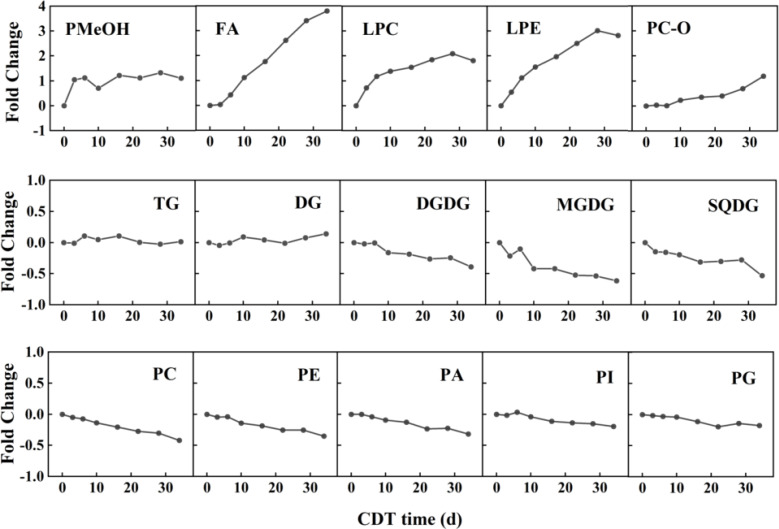
Fold changes in lipids of aged safflower seeds. The fold change was calculated as the mean (n = 3) of each class of total lipids using the formula (A2-A1)/A1, where A2 is the total amount of lipid class represented at the specified aging time point, and A1 is the total amount of the specified lipid class on day 0 (C0). CDT, controlled deterioration treatment.

For the untargeted lipidome, the greatest changes in metabolites between groups were identified by using variable importance in projection (VIP) and fold change (**Table 2**). For C3 seeds, the content of lipid as phosphatidylmethanol (PMeOH) showed a marked increase, from 6.98 to 14.29 nmol/g (105%). This fold-change value above two was maintained until the end of aging (**Figure 3**). From the 10th day of CDT (i.e., C10 onward), the FAs also showed a significant accumulation. FA levels from C3–C34 were 1,282, 1,766, 2,611, 3,416, 4,461, 5,449, and 5,928 nmol/g, representing increases of 4%, 43%, 111%, 176%, 261%, 341%, and 379%, respectively, compared with the control. FA18:2 accounted for the most of the total FA content and increased the most intensely (**Figure S3**). The pattern in FA18:2 and FA20:1 were found to be significantly different starting from C3. Another top changes in lipids classes were the lysophospholipids (LPC and LPE) whose contents significantly rise in a parabolic fashion from the beginning to the end of aging. In particular, LPC18:2 and LPE 18:2 had a fold-change value above 2 from the sixth day of CDT.

**Table 2:** Screening list of lipid differential metabolites.

Compounds	Class	Formula	Adduct type	m/z	C3	C6	C10	C16	C22	C28	C34
FA 16:0	FA	C16H32O2	[M−H]−	255.23386	–	–	Up	Up	Up	Up	Up
FA 18:0	FA	C18H36O2	[M−H]−	283.26544	–	–	–	–	–	Up	Up
FA 18:1	FA	C18H34O2	[M−H]−	281.2511	–	–	Up	Up	Up	Up	Up
FA 18:2	FA	C18H32O2	[M−H]−	279.23361	–	Up	Up	Up	Up	Up	Up
FA 20:1	FA	C20H38O2	[M−H]−	309.28067	–	Up	Up	Up	Up	Up	Up
FA 22:0	FA	C22H44O2	[M−H]−	339.32809	–	–	–	Up	Up	Up	Up
FA 24:0	FA	C24H48O2	[M−H]−	367.35968	–	–	–	Up	–	Up	Up
LPC 16:0	LPC	C24H50NO7P	[M+CH3COO]−	554.34943	–	Up	Up	Up	Up	Up	Up
LPC 18:0	LPC	C26H54NO7P	[M+CH3COO]−	582.37817	–	Up	Up	Up	Up	Up	Up
LPC 18:1	LPC	C26H52NO7P	[M+CH3COO]−	580.36456	Up	Up	Up	Up	Up	Up	Up
LPC 18:2	LPC	C26H50NO7P	[M+CH3COO]−	578.34552	Up	Up	Up	Up	Up	Up	Up
LPE 16:0	LPE	C21H44NO7P	[M−H]−	452.27887	–	Up	Up	Up	Up	Up	Up
LPE 18:0	LPE	C23H48NO7P	[M−H]−	480.30765	–	–	–	Up	Up	Up	Up
LPE 18:1	LPE	C23H46NO7P	[M−H]−	478.2966	–	Up	Up	Up	Up	Up	Up
LPE 18:2	LPE	C23H44NO7P	[M−H]−	476.27838	Up	Up	Up	Up	Up	Up	Up
PC O-43:3|PC O-24:2_19:1	PC	C51H98NO7P	[M+CH3COO]−	926.71979	–	–	–	–	–	Up	Up
PMeOH34:2|PMeOH 16:0_18:2	PMeOH	C38H71O8P	[M−H]−	685.47937	Up	Up	Up	Up	Up	Up	Up
PMeOH36:3|PMeOH 18:1_18:2	PMeOH	C40H73O8P	[M−H]−	711.49426	Up	Up		Up	Up	Up	Up
PMeOH36:4|PMeOH 18:2_18:2	PMeOH	C40H71O8P	[M−H]−	709.48602	Up	Up	Up	Up	Up	Up	Up
DG 32:3|DG 14:1_18:2	DG	C35H62O5	[M+NH4]+	580.48462	–	–	Up	Up	Up	Up	Up
DG 34:4|DG 16:2_18:2	DG	C37H64O5	[M+NH4]+	606.49976	–	–	–	–	–	Up	Up
DG 35:4|DG 17:2_18:2	DG	C38H66O5	[M+NH4]+	620.51697	–	–	–	–	–	–	Up
TG 64:4|TG 18:1_28:1_18:2	TG	C67H122O6	[M+NH4]+	1040.9585	–	Up	–	–	–	–	–
TG 66:4|TG 24:1_24:1_18:2	TG	C69H126O6	[M+NH4]+	1068.99622	–	Up	–	–	–	–	–
PC 34:1|PC 16:0_18:1	PC	C42H82NO8P	[M+CH3COO]−	818.5979	–	–	–	–	–	–	Down
PC 36:1|PC 18:0_18:1	PC	C44H86NO8P	[M+CH3COO]−	846.633	–	–	–	–	–	–	Down
PC 36:3|PC 18:1_18:2	PC	C44H82NO8P	[M+CH3COO]−	842.5954	–	–	–	–	–	–	Down
PE 32:0|PE 16:0_16:0	PE	C37H74NO8P	[M−H]−	690.50806	–	–	–	–	–	–	Down
PE 35:2|PE 17:0_18:2	PE	C40H76NO8P	[M−H]−	728.53058	–	–	–	–	Down	Down	Down
MGDG 36:4|MGDG 18:2_18:2	MGDG	C45H78O10	[M+NH4]+	796.5918	–	–	–	Down	Down	Down	Down
SQDG 34:2|SQDG 16:0_18:2	SQDG	C43H78O12S	[M+NH4]+	836.55273	–	–	–	–	–	–	Down
SQDG 36:2|SQDG 18:0_18:2	SQDG	C45H82O12S	[M+NH4]+	864.58795	–	–	–	–	–	–	Down
SQDG 36:3|SQDG 18:1_18:2	SQDG	C45H80O12S	[M+NH4]+	862.57275	–	–	–	–	–	–	Down
SQDG 36:4|SQDG 18:2_18:2	SQDG	C45H78O12S	[M+NH4]+	860.55658	–	–	–	–	–	–	Down

C3, C6, C10, C16, C22, C28, and C34 represent seeds aged for 3, 6, 10, 16, 22, 28, and 34 days, respectively. Up, upregulation; Down, downregulation; -, no significant difference. Compared with the control (C0), the screening conditions are as follows: (1) fold change ≥ 1.5 or fold change ≤ 0.5. (2) On the basis of the above, select metabolites with VIP ≥ 1.

The seed glycerolipids, including MGDG, DGDG, and SQDG, showed a decreasing trend from the beginning of CDT, until the 16th day of aging (C16). MGDG was screened as a significant changing lipid with a VIP value of 1.55 and a fold-change value of 0.48. At the end of aging (i.e., C34), all SGDG had significant reduction. All the phospholipids, including PE, PC, PI, PG, and PA, showed a decreasing trend with aging, with PE18:2 significantly different from the 22nd day of the CDT. Even the PA showed a slow decreasing trend with seed aging.

### Change of enzymatic activity

**Figure 4:**
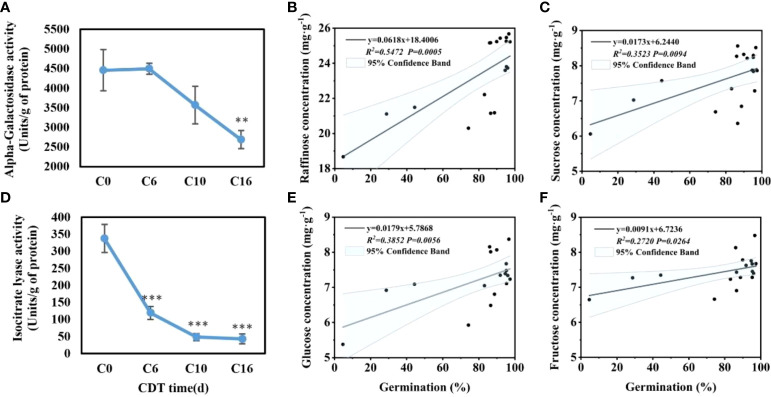
Effects of aging days on the activity of alpha-galactosidase **(A)** and isocitrate lyase **(D)**, and the correlation between germination and concentration of raffinose **(B)**, glucose **(E)**, sucrose **(C)**, and fructose **(F)**. Data are mean ± SE. * indicates a significant difference at P < 0.05, ** at P < 0.01, *** at P < 0.001

Because raffinose is significantly reduced near C10, the safflower seeds were sampled at C0, C6, C10, and C16 to evaluate the activity of α-GAL (**Figure 4A**). With increasing aging time, seed α-GAL activity decreased, reaching a significant change at 16 days of aging. The α-GAL activity of C10 decreased greatly, which was not significant compared with C6 and C16. From C16 to C10, enzyme activities were decreased by 24.62%.

The ICL activity in imbibed seeds was significantly downregulated on aging (**Figure 4B**). For unaged safflower seeds, ICL activity was at the highest level of 337.59 U/g, whereas the activity appeared to be significantly reduced starting at C3, which may indicate that the glyoxylic acid cycle was not functioning properly.

### Correlation between sugar content and germination rate

The pattern of decrease in soluble sugar content was similar to the decrease in seed viability (**Figure 1A**). Therefore, the correlation analysis was made between the change of the sugar content and the change of the germination level (**Figures 4C–F**). Through Pearson correlation analysis, it was found that the seed germination rate was significantly positively correlated with the contents of the four sugars. Raffinose had the largest correlation coefficient of 0.5472. The correlation coefficients of glucose, sucrose, and fructose with aging were 0.3852, 0.3523, and 0.2720, respectively.

### The change to the cell membrane structure

**Figure 5:**
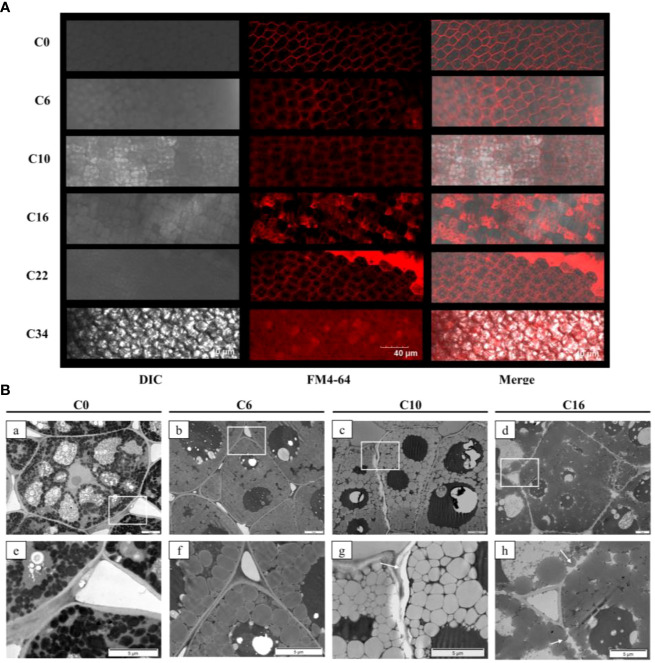
Effects of aging on the integrity of the plasma membrane of safflower seeds. **(A)** Changes in cell membrane permeability of safflower seeds with increasing aging days. Bars = 40 μm. **(B)** Ultrastructure of cotyledon tissues and cell wall complex from C0 [**(a, e)**], C6 [**(b, f)**], C10 [**(c, g)**], and C16 [**(d, h)**]. The white box in the first column diagram represents the enlarged position of the second column in the first column. White arrows indicate the location of cell damage. Bars = 5 μm.

The cell membrane and cytoplasm were stained by FM4-64, which is a membrane dye that can specifically bind to the plasma membrane and endomembrane organelles and emit high-intensity fluorescence (Parlanti et al., 2011). As a specific indicator of membrane integrity, FM4-64 can be used to judge the degree of damage to the cell membrane. Cell membrane outlines were clearly discernible in control (C0) seeds, whereas the membrane structures of oil bodies inside the cells of C10 seeds were stained, but not darkly (**Figure 5A**). The intracellular fluorescence gradually increased with the decreasing seed viability. Most of the cell membrane profiles were not well distinguishable in C34 seeds. TEM was used to study ultrastructural changes in the plasma membrane and cell wall complexes of C0, C6, C10, and C16 cotyledons (**Figure 5B**). The organelles in C0 cells were clearly visible, and the cell wall and plasma membrane structure were intact. Compared with the oil bodies in C0 seeds, those in C10 seeds were largely decomposed, and the cell walls were broken. The C16 seeds cell wall showed massive fragmentation, irregular packing of the plasma membrane, and the existence of a gap between the plasma membrane and the secondary cell wall, indicating that the structural changes of the plasma membrane occurred during the aging process. Greater structural changes and metabolism of oil bodies occurred in the three aged seeds compared with C0.

### Seed vigor and seedling establishment

**Figure 6:**
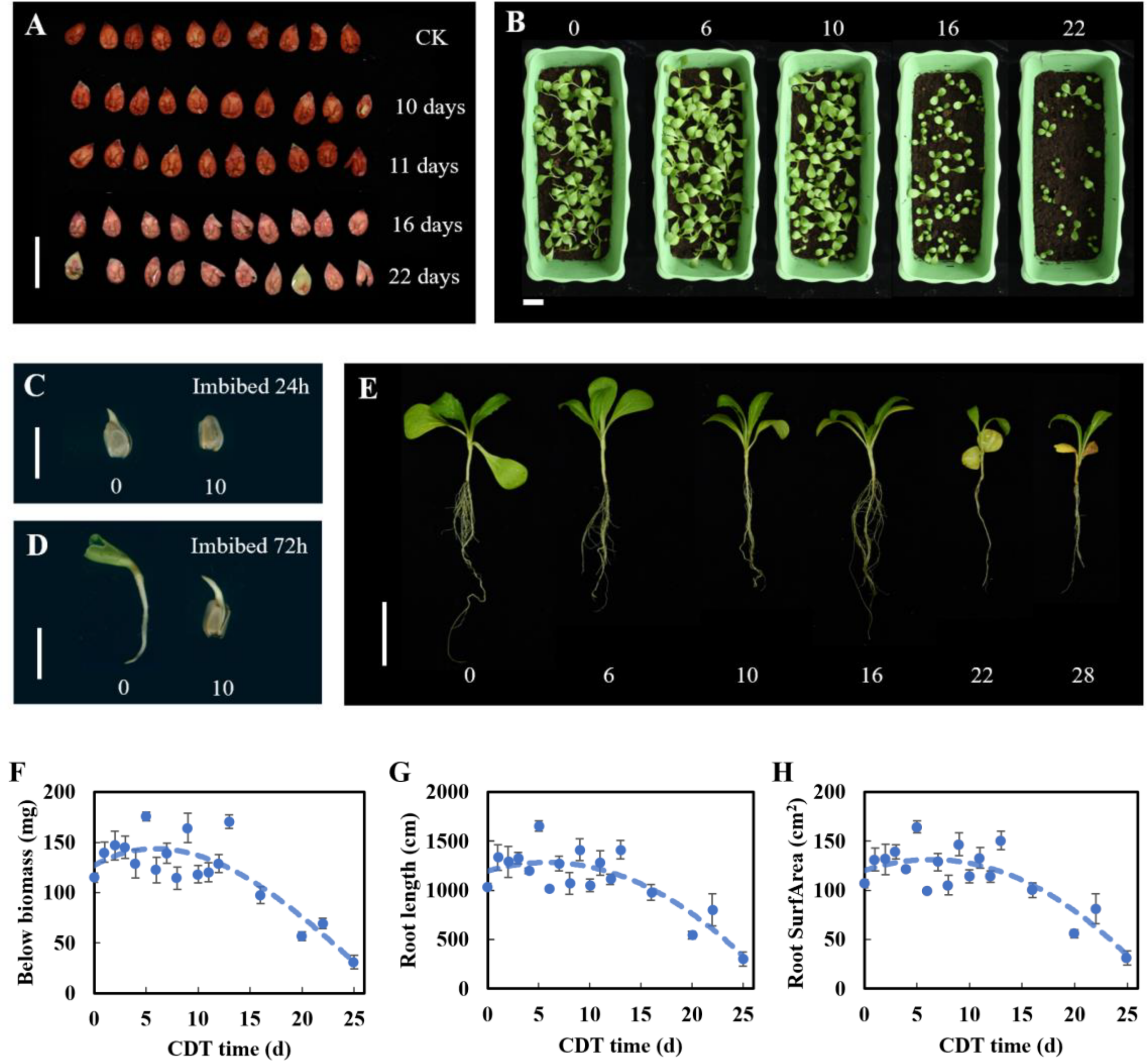
Effect of aging on seed vigor and seedling establishment. **(A)** Seed vigor. Scale bar = 10 mm. **(B)** Seedling phenotype (15 days after sowing). Scale bar = 50 mm. **(C, D)** The growth of C0 and C10 seeds after 24 and 72 h of imbibition. Scale bar = 10 mm. **(E)** Growth of seedlings with different aging times. Scale bar = 50 mm. **(F–H)** Root biomass, root length, and root surface area of seedlings.

Previous studies have demonstrated that 2,3,5-triphenyltetrazolium chloride staining can characterize cell viability (Zhou et al., 2021). Therefore, we studied the effect of aging time on seed viability and found that the seed cell vital staining was significantly lower than that of the control after 16 days of aging (**Figure 6A**). Next, we observed the quality of seedlings at approximately C10 and C16. Seedlings formed after 15 days from sowing (**Figures 6B–E**). With the increase in aging days, the number of growing seedlings decreased and was obviously dwarfed when grown from seeds aged for 16 days (C16). Taking 10 grown seedlings as a replicate, the root system analysis showed that root DW, root length, and root surface area all decreased with increasing aging days from C16 (**Figures 6F–H**). On the basis of the change in the germination level, seed vigor, seedling phenotype, and root parameters, some seeds did not support seedling formation after 16 days of aging.

## Discussion

### Safflower seeds subjected to controlled deterioration (50°C and 60% RH) showed a critical node

Previous studies have provided important evidence that the CN exists during seed deterioration that can serve as trigger for germplasm regeneration (Dulloo et al., 2008). For example, in wheat seeds, the plateau period of an ST genotype is longer than that of an SS genotype, and ST seeds have a higher activity in metabolic processes, protein synthesis, transcription, cell growth/division, and signal transduction (Chen et al., 2021). The reduced energy metabolism in rice seeds at the CN resulted in decreased antioxidant capacity, whereas seed storage proteins were upregulated and carbonylated, indicating a lower ability of seeds to utilize seed storage proteins for germination compared with before the CN (Yin et al., 2016). Seed bank operators take 85% of the initial germination as the decision threshold for seed regeneration, which coincides with the CN on the seed survival curve (Yin et al., 2017). Because of differences in seed life span between species and within species, the germination level of the CN could vary but are, generally, approximately 85% (Guzzon et al., 2021). For oil crops, large amounts of lipids increase the risk of seed deterioration during storage, and oil content can have a large impact on the absolute life span (Nagel and Börner, 2010). Our findings are consistent with previous studies such that the survival curve of safflower seeds presents an inverted S shape during the CDT (Yin et al., 2016). According to the simulation curve, an obvious inflection point in the safflower seed survival curve indicates that the aging process can be separated into two phases: a plateau phase and a more rapid aging phase. On the basis of changes in germination through simulated curves and different imbibition time points, we found that the CN of safflower seeds is near C10 (i.e., day 10 of aging) when the germination level is 86%. When the germination level was below 86%, the quality and the anti-aging properties of the seeds decreased significantly, indicating the need for seed regeneration for banking.

Although the CNs of different crops are all at approximately 85% germination rate, the time to reach the CN differs with the conditions of the CDT (Nagel and Börner, 2010) as temperature and humidity are important external environmental factors that determine seed life span (Zinsmeister et al., 2020). In such studies on grain crops, rice, and wheat, the aging conditions were either natural aging or a CDT at 40°C and 75% RH (Yin et al., 2017; Chen et al., 2019). In general, the higher the RH, the shorter the seed aging time (Lehner et al., 2008; Balešević-Tubić et al., 2010). Although safflower had lower RH (60% RH) compared with the aging condition of wheat (75%RH), the time to reach the CN was shorter, suggesting that safflower seeds were more susceptible to aging than wheat. The wheat seeds of the aging-sensitive genotype reached a CN at 18 days of aging (Chen et al., 2021), whereas the safflower seeds treated with controlled deterioration (50°C and 60% RH) showed a CN at approximately 10 days of aging. Such interspecies differences in seed life span are well known (Colville and Pritchard, 2019).

### Cell energy and structure play a decisive role in germination before and after CN of safflower seed

Levels of energy storage and metabolism are known to be directly and positively correlated with seed longevity (Mao and Sun, 2015; Penfield, 2017; Zhou et al., 2019). The main energy sources used by seeds during germination and seedling formation are soluble sugars, mainly sucrose and fructose (Graham, 2008). In the early stage of germination, soluble carbohydrates stored internally are first consumed, and then, lipids are metabolized into sugars to complete germination and seedling formation (Erbaş et al., 2016; Sinha et al., 2020). Long-term storage is known to cause the loss of a large number of nutrients in seeds, mainly carbohydrates and proteins (Saxena et al., 2010). Seed aging under CDT causes the hydrolysis of soluble sugars, reducing the sugar content (Nigam et al., 2019). During the aging process of corn seeds, the content of raffinose and sucrose in seed embryos decreases steadily (Bernal-Lugo and Leopold, 1992). In addition, lipids are closely linked to seed longevity (Oenel et al., 2017). Fully acylated and unoxidized storage lipids (such as triacylglycerols) and structural lipids (such as phospholipids and galactolipids) in wheat seeds are positively associated with seed longevity, whereas a large number of oxidative variants and hydrolysates (such as lysophospholipids and FAs) are inversely associated with longevity (Riewe et al., 2017; Wiebach et al., 2020).

We first measured the change of direct energy sugar content with aging time and showed that the level of sucrose in safflower seeds gradually decreases, which is similar to other aging seeds (Huang et al., 2021). From the germination levels of seeds after different aging times, it is evident that the seeds of C6 and C10 germinate slower, which is related to the reduction of direct energy. The final germination levels of C6 and C10 did not decrease significantly, though, indicating that energy was still available for conversion into usable sugars for germination (Bhattacharya et al., 2015). In oilseeds, lipids are important energy storage substances, and we found that the content of lipid hydrolysates, such as LPC, LPE, and FA, increases significantly as aging progresses from C3; especially, FAs increased the most before C10. While the sucrose decreases slowly, the FA content in the seeds accumulates a lot; for example, the FA content in the C6 seeds is 1.43 times that of the C0 seeds. Oilseeds convert fats into carbohydrates through the glyoxylic acid cycle and other processes during germination (Faraoni et al., 2019). ICL is one of the key enzymes of the glyoxylate cycle (Lee et al., 2020). Its activity in safflower seeds is inhibited as aging time increases, although it still has a certain activity at C6. Therefore, FAs can generate sucrose through these processes. Although the speed of germination of the C6 seed is slower than that of the C0 seed, the germination level is not lower. Thus, energy metabolism in the plateau phase of the seed population survival curve determines seed germination efficiency before the vigor of the seeds drops abruptly.

Unlike the slow downward trend of sucrose, we found that raffinose, fructose, and glucose contents did not change much before C10 but dropped abruptly at the CN. Generally speaking, soluble carbohydrates are thought to be involved in desiccation tolerance and seed storability (Garcia et al., 2006). High levels of raffinose family oligosaccharides have been correlated with seed longevity (Bailly et al., 2001; ElSayed et al., 2014; Lee et al., 2019). For example, the raffinose content of viable seeds in corn seeds is higher than that of non-viable seeds (Castillo et al., 1990). In dry seeds, sucrose and raffinose act as a replacement for water, maintaining the hydrogen bonds required for membrane and protein stabilization (ElSayed et al., 2014), which are protectors of cell membrane homeostasis (Caffrey et al., 1988). During germination, raffinose in some seed cotyledons is directly hydrolyzed to produce sucrose to facilitate germination (Kuo et al., 1988; Dierking and Bilyeu, 2009). Through correlation analysis, we found that four sugars had a significant positive correlation with the germination level, especially raffinose. To understand the reason for the decline in raffinose, the enzyme activity that catabolizes it was measured. α-GAL is a glycoside hydrolase responsible for processing raffinose family oligosaccharides (RFOs) into metabolizable sugars (Arunraj et al., 2020). RFOs are the major natural substrates of α-GAL (Jang et al., 2019; Bhatia et al., 2020). The results showed that the α-GAL enzyme activity that decomposes raffinose decreases with aging time, especially after the CN. Therefore, seed senescence also had a significant effect on enzymatic activity, although it was not possible to determine whether it was involved in the reduction of raffinose concentration. The drop in raffinose at the CN may indicate a disruption of the cytoplasmic glass state, which is closely related to an acceleration of seed aging (Ballesteros et al., 2020).

The aging process of seeds includes damage to the plasma membrane in addition to energy consumption (Ebone et al., 2019), whereas the node at which the plasma membrane is damaged is poorly understood (Buijs et al., 2020). We showed that the FAs at the nodes are continuously hydrolyzed, whereas the safflower seed germination level is significantly reduced, indicating that energy metabolism is no longer the key to seed germination. The sugar and lipid results have shown a strong response to aging during the plateau period, and the sugar-decreasing node at this point indicates that the seed’s resistance to aging has entered another stage. We hypothesize that damage to the plasma membrane at this time is the dominant factor in seed germinability, as the structural lipid content is significantly reduced at the node and beyond. The analysis showed that PC, PE, and PI are significantly reduced at C10, and that the contents of PA and PG are significantly reduced at C16. It shows that phospholipids considerably decrease with the deepening of aging, especially after C10. The aging process of seeds is accompanied by lipid oxidation, which triggers the hydrolysis of storage and structural lipids and leads to loss of seed vigor (Wiebach et al., 2020). Because membranes are mainly composed of phospholipids and proteins, the disappearance of either from the cell can lead to a reduction in the area where the membrane exists. Loss of phospholipids allows solutes to ooze out of the cells. Thus, the substantial decrease in viability after C10 coincides with the decrease in phospholipids at that time.

In addition to phospholipids, glycolipids are closely related to cell structure. The MGDG and DGDG are well known as the most abundant lipids of photosynthetic membranes (thylakoids) (Gabruk et al., 2017). These galactolipids are directly involved in the photosynthetic reaction in higher plants and cyanobacteria for they are key elements in determining the functionality of the thylakoid membrane. Galactolipids are believed to play essential roles in the light-dependent conversion of prolamellar bodies to thylakoid membranes in germinating seeds (Fujii et al., 2017; Rocha et al., 2018). In severely aged safflower seeds, the germination level is less than 70%, the glycolipids are largely destroyed, and their content significantly decreases significantly in the later stage of aging.

As a validation, we recorded the cellular microstructure of aged seeds with different germination levels, with both confocal microscopy (Goto-Yamada et al., 2019) and TEM (Lee et al., 2012). By staining with FM4-64, we found that the dye slowly penetrates into the cell interior as the aging time increases, suggesting that the permeability of the cell membrane changes. Changes in the structure of individual cells can be clearly seen from the TEM, such that the C10 seed cell wall structure is brittle and shows obvious signs of plasmolysis. At C16, the cell wall and plasma membrane structures are disrupted. In addition, the seedling phenotype also shows changes at the CN for seed viability.

The above results indicate that, with the deepening of safflower seed aging, the metabolic capability of cells gradually decreases and the integrity of cell membranes is destroyed. At the point of the CN, the cell structure obviously began to show damage, which led to some seeds not being able to germinate normally and other seeds germinating with an obvious lag. The division of seed population deterioration into two phases above and below the CN enabled the biochemical signature of the phases of aging to be revealed. At the CN, the main factor determining seed germination at the node shifts from energy metabolism to structural impairment. We propose that, as the CN is approached, the reduction of sugars that maintain the cells’ glass state and the destruction of structural lipids of the plasma membrane destabilize the seed. Changes in seed storage compounds and plasma membranes at the CN result in irreversible structural damage and loss of biochemical competences, facilitating the loss of seeds’ normal function.

### Research prospect of repair mechanism before the critical node

It is generally accepted that there are protective and repair mechanisms inside the aged seeds (Rajjou and Debeaujon, 2008), and it is known that the enhancement of seed vigor and the successful priming rely on DNA repair mechanisms activated during imbibition (Ventura et al., 2012). The germination levels before reaching C10 in this experiment remained high, suggesting that it is possible for the metabolism to activate once the seeds are imbibed and damage can be repaired, thereby improving seed vigor. Nevertheless, even with protective mechanisms, e.g., the formation of glassy cytoplasm and the production of antioxidants (Jeevan Kumar et al., 2015), and repair after imbibition through enzymes such as DNA glycosylase and methionine sulfoxide reductase (Waterworth et al., 2010; Waterworth et al., 2015), mature dry seeds gradually accumulate cellular damage during aging (Sano et al., 2016). This means that the protection and repair of seeds have limitations.

In addition, because of the high-temperature aging conditions, seed internal substances respond to high-temperature stress. The ratio of PC/PE has been related to differences in seed desiccation tolerance (Chen et al., 2018). The content of lysophospholipids began to increase significantly in the early stage of aging. The phospholipids PC and PE of various carbon chain lengths gradually decrease and are decomposed in the early stages of aging (**Table 2**). At a high temperature, the PC/PE ratio in wheat pollen increases significantly, which suggests that this metabolic change is a compensation for the physical changes in membrane structure caused by heat and that lipid remodeling is an adaptive mechanism under high-temperature stress (Narayanan et al., 2018; Higashi and Saito, 2019). For safflower seeds aged 0–10 days, a large amount of PC hydrolysis is manifested as a significant increase in LPC and the maintenance of a high ratio of PC/PE. This indicates that lipid remodeling is in progress during the early stages of aging, whereas in the later stage of aging, the changes in lipid composition could no longer resist aging stress.

Finally, PMeOH is newly reported as a rare phospholipid class characterized in *Euglena gracilis* whose detection is now possible because of the updated detection technology (Tsugawa et al., 2019). Increased and accurate annotation of lipids means that we found PMeOH in this experiment and identified it as a significant difference marker as it increases in aged safflower seeds. This substance may act as an intermediate metabolite of safflower in the early stage of aging.

## Conclusions

**Figure 7:**
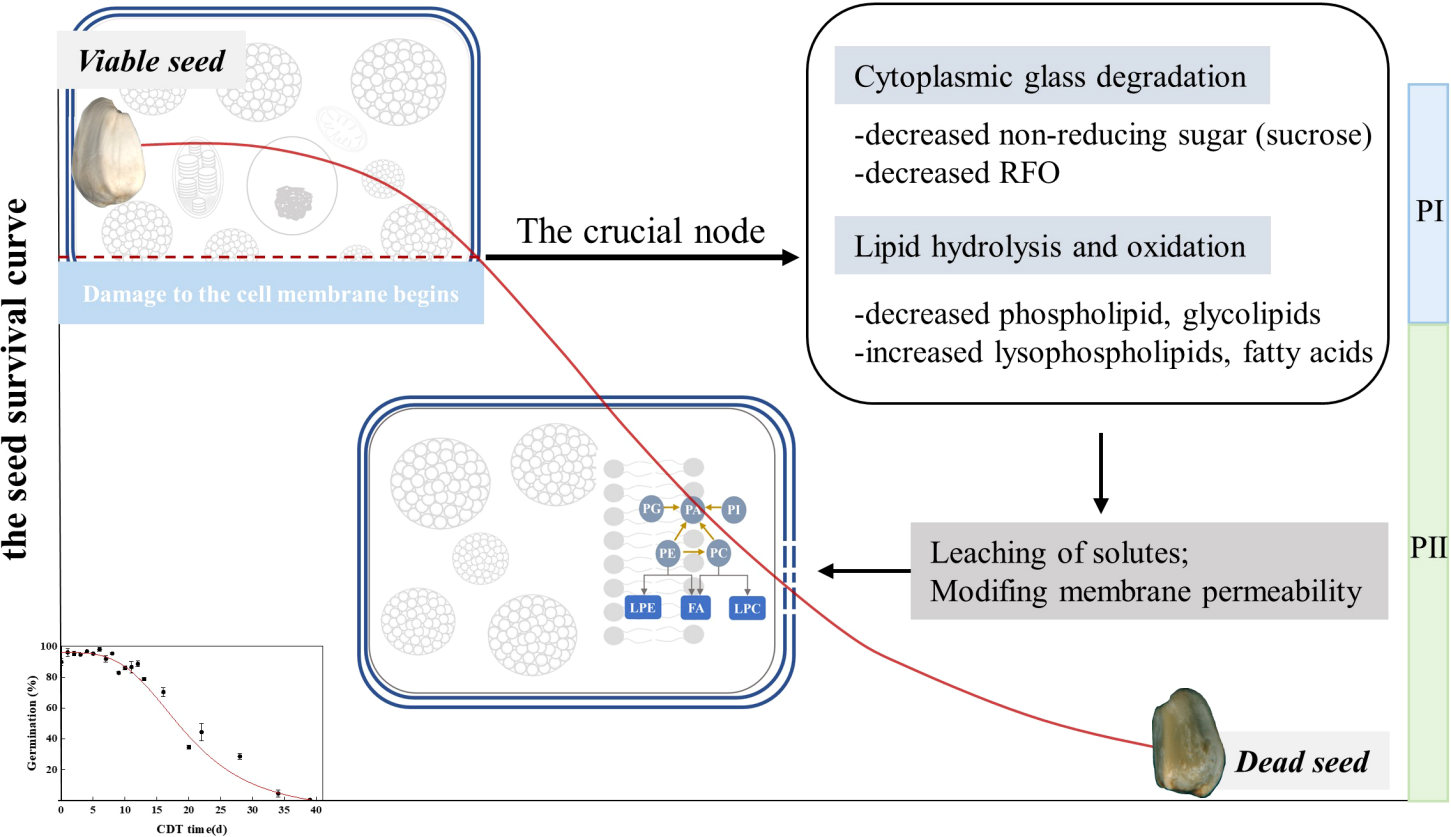
Schematic diagram of the proposed model showing the seed survival curve of an enlarged version of safflower. The seeds gradually lost their vigor with increasing aging time. In the PI phase, seed lipids and carbohydrates undergo energy metabolism. At the CN, the direct energy substance, sugar, is low, and structural lipids are hydrolyzed and oxidized. The structure of the seed cell membrane begins to be destroyed, which brings irreversible damage to the seed vigor. During the PII period, a large amount of lipid hydrolysates accumulated and could not be metabolized, resulting in delayed seed germination and inhibition of seedling formation. Aging eventually made seeds unable to germinate.

We designed the CDT artificial aging experiment of safflower seeds and obtained the survival curve of the seeds (**Figure 7**). The results show a CN around the germination level of 86%. Before the CN, various materials such as lipids and sugars were metabolized to resist accelerated aging. After the CN, the soluble sugar content is reduced, likely affecting cytoplasmic vitrification. Under the condition that the content of the main storage lipid triacylglycerol did not change significantly with aging time, the hydrolysis of phospholipids and the massive accumulation of FAs indicated that the stability of the cell membrane structure is destroyed. This eventually leads to the decrease in the seed vigor, which became an irreversible process. In conclusion, this study reveals that changes in seed cell metabolism and the damage to the cell membrane structure beyond the CN are the main reasons for the sharp decline of cell viability. This experiment provides a scientific basis for the need for the renewal of oilseeds preserved in the germplasm bank before seed germination falls far below 85%.

## Data availability statement

The raw data supporting the conclusions of this article will be made available by the authors, without undue reservation.

## Author contributions

TZ, JP and JY defined the research theme and wrote the manuscript. LZ, LL and CC designed methods and experiments, carried out the laboratory experiments, analyzed the data, and interpreted the results. QW, FW, JC, HP and CP co-designed the experiments and discussed the analyses and interpretation. All authors contributed to the article and approved the submitted version.

## Funding

The work was financially supported by the China Postdoctoral Science Foundation (2020M673566XB), the Science and Technology Department of Sichuan Province (2019YJ0333, 2021YFYZ0012-5), the National Natural Science Foundation of China (81503200), and Innovation Team and Talents Cultivation Program of National Administration of Traditional Chinese Medicine (ZYYCXTD-D-202209).

## Conflict of interest

The authors declare that the research was conducted in the absence of any commercial or financial relationships that could be construed as a potential conflict of interest.

## Publisher’s note

All claims expressed in this article are solely those of the authors and do not necessarily represent those of their affiliated organizations, or those of the publisher, the editors and the reviewers. Any product that may be evaluated in this article, or claim that may be made by its manufacturer, is not guaranteed or endorsed by the publisher.

## References

[B1] Ahmad M. Waraich E. A. Skalicky M. Hussain S. Zulfiqar U. Anjum M. Z.. (2021a). Adaptation strategies to improve the resistance of oilseed crops to heat stress under a changing climate: An overview. Front. Plant Sci. 12, 767150. doi: 10.3389/fpls.2021.767150 34975951 PMC8714756

[B2] Ahmad M. Waraich E. A. Tanveer A. Anwar-ul-Haq M. (2021b). Foliar applied thiourea improved physiological traits and yield of camelina and canola under normal and heat stress conditions. J. Soil Sci. Plant Nutr. 21, 1666–1678. doi: 10.1007/s42729-021-00470-8

[B3] Ahmad M. Waraich E. A. Usman Z. Aman U. Muhammad F. (2021c). Thiourea application improves heat tolerance in camelina (Camelina sativa l. crantz) by modulating gas exchange, antioxidant defense and osmoprotection. Ind. Crops Products 170, 113826. doi: 10.1016/j.indcrop.2021.113826

[B4] Amini H. Ahmad A. Mostafa K. (2014). Effect of water deficiency on seed quality and physiological traits of different safflower genotypes. Turkish J. Biol. 38, 271–282. doi: 10.3906/biy-1308-22

[B5] Argosubekti N. (2020). “A review of heat stress signaling in plants,” in IOP Conference Series: Earth and Environmental Science. 484, 012041 (Makassar City, Indonesia: IOP Publishing).

[B6] Arunraj R. Skori L. Kumar A. Hickerson N. M. N. Shoma N. Vairamani M.. (2020). Spatial regulation of alpha-galactosidase activity and its influence on raffinose family oligosaccharides during seed maturation and germination in cicer arietinum. Plant Signal Behav. 15, 1709707. doi: 10.1080/15592324.2019.1709707. 31906799 PMC8570745

[B7] Bailly C. (2004). Active oxygen species and antioxidants in seed biology. Seed Sci. Res. 14, 93–107. doi: 10.1079/SSR2004159

[B8] Bailly C. Audigier C. Ladonne F. Wagner M. H. Coste F. Corbineau F.. (2001). Changes in oligosaccharide content and antioxidant enzyme activities in developing bean seeds as related to acquisition of drying tolerance and seed quality. J. Exp. Bot. 52, 701–708. doi: 10.1093/jexbot/52.357.701 11413206

[B9] Balešević-Tubić S. Tatić M. Ðorđević V. Nikolić Z. Ðukić V. (2010). Seed viability of oil crops depending on storage conditions. Helia 33, 153–160. doi: 10.2298/HEL1052153B

[B10] Ballesteros D. Hill L. M. Lynch R. T. Pritchard H. W. Walters C. (2019). Longevity of preserved germplasm: the temperature dependency of aging reactions in glassy matrices of dried fern spores. Plant Cell Physiol. 60, 376–392. doi: 10.1093/pcp/pcy217 30398653

[B11] Ballesteros D. Pritchard H. W. Walters C. (2020). Dry architecture: Towards the understanding of the variation of longevity in desiccation-tolerant germplasm. Seed Sci. Res. 30(2), 142–155. doi: 10.1017/S0960258520000239

[B12] Bernal-Lugo I. Leopold A.C. (1992). Changes in soluble carbohydrates during seed storage. Plant Physiol. 98, 1207–1210. doi: 10.1104/pp.98.3.1207 16668748 PMC1080329

[B13] Bewley J. D. Bradford K. Hilhorst H. (2012). Seeds: physiology of development, germination and dormancy (Berlin, Germany: Springer Science & Business Media).

[B14] Bhatia S. Singh A. Batra N. Singh J. (2020). Microbial production and biotechnological applications of alpha-galactosidase. Int. J. Biol. Macromol 150, 1294–1313. doi: 10.1016/j.ijbiomac.2019.10.140 31747573

[B15] Bhattacharya S. Chowdhury R. Mandal A. K. (2015). Seed invigoration treatments for improved germinability and field performance of soybean [Glycine max (L.) merill]. Indian J. Agric. Res. 49, 32–38. doi: 10.5958/0976-058X.2015.00004.9

[B16] Buijs G. Willems L. A.J. Kodde J. Steven P. C. Groot S. Bentsink L. (2020). Evaluating the EPPO method for seed longevity analyses in arabidopsis. Plant Sci. 301, 110644. doi: 10.1016/j.plantsci.2020.110644 33218622

[B17] Caffrey M. Fonseca V. Leopold A.C. (1988). Lipid-sugar interactions: relevance to anhydrous biology. Plant Physiol. 86, 754–758. doi: 10.1104/pp.86.3.754 16665982 PMC1054564

[B18] Castillo E. M. De Lumen B. O. Reyes P. S. De Lumen H. Z. (1990). ‘Raffinose synthase and galactinol synthase in developing seeds and leaves of legumes’, J. Agricultural Food Chem. 38, 351–355. doi: 10.1021/jf00092a003

[B19] Chen X. Borner A. Xin X. Nagel M. He J. Li J.. (2021). Comparative proteomics at the critical node of vigor loss in wheat seeds differing in storability. Front. Plant Sci. 12, 707184. doi: 10.3389/fpls.2021.707184 34527008 PMC8435634

[B20] Chen B. Yin G. Whelan J. Zhang Z. Xin X. He J.. (2019). Composition of mitochondrial complex I during the critical node of seed aging in oryza sativa. J. Plant Physiol. 236, 7–14. doi: 10.1016/j.jplph.2019.02.008 30840921

[B21] Chen H. Yu X. Zhang X. Yang L. Huang X. Zhang J.. (2018). Phospholipase Dalpha1-mediated phosphatidic acid change is a key determinant of desiccation-induced viability loss in seeds. Plant Cell Environ. 41, 50–63. doi: 10.1111/pce.12925 28152567

[B22] Colville L. Bradley E. L. Lloyd A. S. Pritchard H. W. Castle L. Kranner I. (2012). Volatile fingerprints of seeds of four species indicate the involvement of alcoholic fermentation, lipid peroxidation, and maillard reactions in seed deterioration during ageing and desiccation stress. J. Exp. Bot. 63, 6519–6530. doi: 10.1093/jxb/ers307 23175670 PMC3504501

[B23] Colville L. Pritchard H. W. (2019). Seed life span and food security. New Phytol. 224(2), 557–562. doi: 10.1111/nph.16006 31225902

[B24] Dajue L Mündel H.-H. (1996). Safflower. Carthamus tinctorius L. Promoting the conservation and use of underutilized and neglected crops. Institute of Plant Genetics and Crop Plant Research, Gatersleben/ International Plant Genetic Resources Institute (Rome, Italy: Bioversity International).

[B25] Davies R. M. Newton R. J. Hay F. R. Probert R. J. (2016). 150-seed comparative longevity protocol a reduced seed number screening method for identifying short-lived seed conservation collections. Seed Sci. Technol. 44, 569–584. doi: 10.15258/sst.2016.44.3.13

[B26] Dierking E. C. Bilyeu K. D. (2009). Raffinose and stachyose metabolism are not required for efficient soybean seed germination. J. Plant Physiol. 166, 1329–1335. doi: 10.1016/j.jplph.2009.01.008 19286275

[B27] Dulloo M. E. Hanson J. Jorge M. A. B. Thormann I. (2008). Regeneration guidelines: general guiding principles. Crop specific regeneration guidelines. Dulloo M.E. Thormann I. Jorge M.A. Hanson J. Eds. (Rome, Italy: CGIAR System-wide Genetic Resource Programme (SGRP)). 6.

[B28] Ebone L. A. Caverzan A. Chavarria G. (2019). Physiologic alterations in orthodox seeds due to deterioration processes. Plant Physiol. Biochem. 145, 34–42. doi: 10.1016/j.plaphy.2019.10.028 31665665

[B29] ElSayed A. I. Rafudeen M. S. Golldack D. (2014). Physiological aspects of raffinose family oligosaccharides in plants: protection against abiotic stress. Plant Biol. 16, 1–8. doi: 10.1111/plb.12053 23937337

[B30] Erbaş S. Tonguç M. Şanli A. (2016). Mobilization of seed reserves during germination and early seedling growth of two sunflower cultivars. J. Appl. Bot. Food Qual. 89, 217–222. doi: 10.5073/JABFQ.2016.089.028

[B31] Faraoni P. Sereni E. Gnerucci A. Cialdai F. Monici M. Ranaldi F. (2019). Glyoxylate cycle activity in pinus pinea seeds during germination in altered gravity conditions. Plant Physiol. Biochem. 139, 389–394. doi: 10.1016/j.plaphy.2019.03.042 30959447

[B32] Fujii S. Kobayashi K. Nagata N. Masuda T. Wada H. (2017). Monogalactosyldiacylglycerol facilitates synthesis of photoactive protochlorophyllide in etioplasts. Plant Physiol. 174, 2183–2198. doi: 10.1104/pp.17.00304 28655777 PMC5543945

[B33] Gabruk M. Mysliwa-Kurdziel B. Kruk J. (2017). MGDG, PG and SQDG regulate the activity of light-dependent protochlorophyllide oxidoreductase. Biochem. J. 474, 1307–1320. doi: 10.1042/BCJ20170047 28188256

[B34] Gao J. Fu H. Zhou X. Chen Z. Luo Y. Cui B.. (2016). Comparative proteomic analysis of seed embryo proteins associated with seed storability in rice (Oryza sativa l) during natural aging. Plant Physiol. Biochem. 103, 31–44. doi: 10.1016/j.plaphy.2016.02.026 26950923

[B35] Garcia I. S. Souza A. Barbedo C. J. Dietrich S. M. C. Figueiredo-Ribeiro R. C. L. (2006). Changes in soluble carbohydrates during storage of caesalpinia echinata LAM.(Brazilwood) seeds, an endangered leguminous tree from the Brazilian Atlantic forest. Braz. J. Biol. 66, 739–745. doi: 10.1590/S1519-69842006000400018 16906306

[B36] Goel A. Sheoran I. S. (2003). Lipid peroxidation and peroxide-scavenging enzymes in cotton seeds under natural ageing. Biol. plantarum 46, 429–434. doi: 10.1023/A:1024398724076

[B37] Goto-Yamada S. Oikawa K. Bizan J. Shigenobu S. Yamaguchi K. Mano S.. (2019). Sucrose starvation induces microautophagy in plant root cells. Front. Plant Sci. 10, 1604. doi: 10.3389/fpls.2019.01604 31850051 PMC6901504

[B38] Graham I. A. (2008). Seed storage oil mobilization. Annu. Rev. Plant Biol. 59, 115–142. doi: 10.1146/annurev.arplant.59.032607.092938 18444898

[B39] Guzzon F. Gianella M. Velazquez Juarez J. A. Sanchez Cano C. E Costich D. (2021). Seed longevity of maize conserved under germplasm bank conditions for up to 60 years. Ann. Bot. 127, 775–785. doi: 10.1093/aob/mcab009 33580665 PMC8103804

[B40] Higashi Y. Saito K. (2019). Lipidomic studies of membrane glycerolipids in plant leaves under heat stress. Prog. Lipid Res. 75, 100990. doi: 10.1016/j.plipres.2019.100990 31442527

[B41] Hong T. D. Ellis R. H. Astley D. Pinnegar A. E. Groot S. P. C. Kraak H. L. (2005). Survival and vigour of ultra-dry seeds after ten years of hermetic storage. Seed Sci. Technol. 33, 449–460. doi: 10.15258/sst.2005.33.2.17

[B42] Huang Y. Cai S. Ruan X. Xu J. Cao D. (2021). CSN improves seed vigor of aged sunflower seeds by regulating the fatty acid, glycometabolism, and abscisic acid metabolism. J. advanced Res. 33, 1–13. doi: 10.1016/j.jare.2021.01.019 PMC8463905 34603775

[B43] Hu D. Ma G. Wang Q. Yao J. Wang Y. Pritchard H. W.. (2012). Spatial and temporal nature of reactive oxygen species production and programmed cell death in elm (Ulmus pumila l.) seeds during controlled deterioration. Plant Cell Environ. 35, 2045–2059. doi: 10.1111/j.1365-3040.2012.02535.x 22582978

[B44] Hussain M.I. Lyra D.-A. Farooq M. Nikoloudakis N. Khalid N. (2016). Salt and drought stresses in safflower: a review. Agron. Sustain. Dev. 36, 1–31. doi: 10.1007/s13593-015-0344-8

[B45] Jang J. M. Yang Y. Wang R. Bao H. Yuan H. Yang J. (2019). Characterization of a high performance alpha-galactosidase from irpex lacteus and its usage in removal of raffinose family oligosaccharides from soymilk. Int. J. Biol. Macromol 131, 1138–1146. doi: 10.1016/j.ijbiomac.2019.04.060 30981775

[B46] Jeevan Kumar S. P. Rajendra Prasad S. Banerjee R. Thammineni C. (2015). Seed birth to death: dual functions of reactive oxygen species in seed physiology. Ann. Bot. 116, 663–668. doi: 10.1093/aob/mcv098 26271119 PMC4578000

[B47] Jumrani K. Bhatia V. S. (2018). Impact of combined stress of high temperature and water deficit on growth and seed yield of soybean. Physiol. Mol. Biol. Plants 24, 37–50. doi: 10.1007/s12298-017-0480-5 29398837 PMC5787112

[B48] Karrfalt R. P. (2008). “'Seed testing', the woody plant seed manual,” in Agriculture handbook, (United States: United States Department of Agriculture. Forest Service) 727, 97–115.

[B49] Knowles P. F. (1980). “Safflower,” in Hybridization of crop plants, (Madison, Wis.: American Society of Agronomy-Crop Science Society of America) 535–548.

[B50] Koutroubas S. D. Papakosta D. K. (2010). Seed filling patterns of safflower: Genotypic and seasonal variations and association with other agronomic traits. Ind. Crops Products 31, 71–76. doi: 10.1016/j.indcrop.2009.09.014

[B51] Kumar S. Ambreen H. T Variath M. R Rao A. Agarwal M. Kumar A.. (2016). Utilization of molecular, phenotypic, and geographical diversity to develop compact composite core collection in the oilseed crop, safflower (Carthamus tinctorius l.) through maximization strategy. Front. Plant Sci. 7, 1554. doi: 10.3389/fpls.2016.01554 27807441 PMC5069285

[B52] Kumari A. (2009). Germination behaviour, viability and longevity of safflower (Carthamus tinctorius l.) seeds. Biosciences 3.

[B53] Kuo T. M. VanMiddlesworth J. F. J Wolf W. (1988). Content of raffinose oligosaccharides and sucrose in various plant seeds. J. Agric. Food Chem. 36, 32–36. doi: 10.1021/jf00079a008

[B54] Lee S. H. Ki D. Kim S. Kim I. K. Kim K. J. (2020). Biochemical properties and crystal structure of isocitrate lyase from bacillus cereus ATCC 14579. Biochem. Biophys. Res. Commun. 533, 1177–1183. doi: 10.1016/j.bbrc.2020.09.136 33041004

[B55] Lee J.-S. Velasco-Punzalan M. Pacleb M. Valdez R. Kretzschmar T. L McNally K.. (2019). Variation in seed longevity among diverse indica rice varieties. Ann. Bot. 124, 447–460. doi: 10.1093/aob/mcz093 31180503 PMC6798842

[B56] Lee J. Welti R. Roth M. Schapaugh W. T. Li J. Trick H. N. (2012). Enhanced seed viability and lipid compositional changes during natural ageing by suppressing phospholipase dalpha in soybean seed. Plant Biotechnol. J. 10, 164–173. doi: 10.1111/j.1467-7652.2011.00650.x 21895945 PMC3728994

[B57] Lehner A. Mamadou N. Poels P. Come D. Bailly C. Corbineau F. (2008). Changes in soluble carbohydrates, lipid peroxidation and antioxidant enzyme activities in the embryo during ageing in wheat grains. J. Cereal Sci. 47, 555–565. doi: 10.1016/j.jcs.2007.06.017

[B58] Lin L. Ma J. Ai Q. Pritchard H. W. Li W. Chen H. (2021). Lipid remodeling confers osmotic stress tolerance to embryogenic cells during cryopreservation. Int. J. Mol. Sci. 22, 2174. doi: 10.3390/ijms22042174 33671662 PMC7926411

[B59] Li W. Wang R. Li M. Li L. Wang C. Welti R.. (2008). Differential degradation of extraplastidic and plastidic lipids during freezing and post-freezing recovery in arabidopsis thaliana. J. Biol. Chem. 283, 461–468. doi: 10.1074/jbc.M706692200 17962199

[B60] Li D. Wang Q. Xu X. Yu J. Chen Z. Wei B.. (2021). Temporal transcriptome profiling of developing seeds reveals candidate genes involved in oil accumulation in safflower (Carthamus tinctorius l.). BMC Plant Biol. 21, 1–17. doi: 10.1186/s12870-021-02964-0 33858333 PMC8051040

[B61] Mani V. Lee S.-K. Yeo Y. Hahn B.-S. (2020). A metabolic perspective and opportunities in pharmacologically important safflower. Metabolites 10, 253. doi: 10.3390/metabo10060253 PMC7344433 32560514

[B62] Mao Z. Sun W. (2015). Arabidopsis seed-specific vacuolar aquaporins are involved in maintaining seed longevity under the control of ABSCISIC ACID INSENSITIVE 3. J. Exp. Bot. 66, 4781–4794. doi: 10.1093/jxb/erv244 26019256 PMC4507774

[B63] Matthews S. Noli E. Demir I. Khajeh-Hosseini M. Wagner M.-H. (2012). Evaluation of seed quality: from physiology to international standardization. Seed Sci. Res. 22, S69–S73. doi: 10.1017/S0960258511000365

[B64] McPherson M. A. Yang R.-C. Good A. G. Nielson R. L. Hall L. M. (2009). Potential for seed-mediated gene flow in agroecosystems from transgenic safflower (Carthamus tinctorius l.) intended for plant molecular farming. Transgenic Res. 18, 281–299. doi: 10.1007/s11248-008-9217-0 18941919

[B65] Nagel M. Börner A. (2010). The longevity of crop seeds stored under ambient conditions. Seed Sci. Res. 20, 1–12. doi: 10.1017/S0960258509990213

[B66] Narayanan S. Prasad P. V. V. Welti R. (2018). Alterations in wheat pollen lipidome during high day and night temperature stress. Plant Cell Environ. 41, 1749–1761. doi: 10.1111/pce.13156 29377219 PMC6713575

[B67] Newton R. Hay F. Probert R. (2014). Protocol for comparative seed longevity testing (Millennium seed bank partnership ardingly). Millennium Seed Bank Partnership, Wakehurst Place, Ardingly, Board of Trustees of the Royal Botanic Gardens, Kew, West Sussex, UK.

[B68] Nigam M. Mishra A. P. Salehi B. Kumar M. Sahrifi-Rad M. Coviello E.. (2019). Accelerated ageing induces physiological and biochemical changes in tomato seeds involving MAPK pathways. Scientia Hortic. 248, 20–28. doi: 10.1016/j.scienta.2018.12.056

[B69] Oenel A. Fekete A. Krischke M. C Faul S. Gresser G. Havaux M.. (2017). Enzymatic and non-enzymatic mechanisms contribute to lipid oxidation during seed agin. Plant Cell Physiol. 58, 925–933. doi: 10.1093/pcp/pcx036 28371855

[B70] Ostberg S. Schewe J. Childers K. Frieler K. (2018). Changes in crop yields and their variability at different levels of global warming. Earth System Dynamics 9, 479–496. doi: 10.5194/esd-9-479-2018

[B71] Parlanti S. Kudahettige N. P. Lombardi L. Mensuali-Sodi A. Alpi A. Perata P.. (2011). Distinct mechanisms for aerenchyma formation in leaf sheaths of rice genotypes displaying a quiescence or escape strategy for flooding tolerance. Ann Bot. 107, 1335–1343. doi: 10.1093/aob/mcr086 21489969 PMC3101152

[B72] Penfield S. (2017). Seed dormancy and germination. Curr. Biol. 27, R874–RR78. doi: 10.1016/j.cub.2017.05.050 28898656

[B73] Pilling D. Bélanger J. Diulgheroff S. Koskela J. Leroy G. Mair G.. (2020). Global status of genetic resources for food and agriculture: challenges and research needs. In Genet. Resour., 1(1), 4–16. doi: 10.46265/genresj.2020.1.4-16

[B74] Probert R. J. Daws M. I. Hay F. R. (2009). Ecological correlates of ex situ seed longevity: a comparative study on 195 species. Ann. Bot. 104, 57–69. doi: 10.1093/aob/mcp082 19359301 PMC2706723

[B75] Pukacka S. Kuiper P. J. C. (1988). Phospholipid composition and fatty acid peroxidation during ageing of acer platanoides seeds. Physiologia Plantarum 72, 89–93. doi: 10.1111/j.1399-3054.1988.tb06627.x

[B76] Rajjou L. Debeaujon I. (2008). Seed longevity: survival and maintenance of high germination ability of dry seeds. Comptes rendus biologies 331, 796–805. doi: 10.1016/j.crvi.2008.07.021 18926494

[B77] Ratajczak E. Małecka A. Bagniewska-Zadworna A. Marzena Kalemba E. (2015). The production, localization and spreading of reactive oxygen species contributes to the low vitality of long-term stored common beech (Fagus sylvatica l.) seeds. J. Plant Physiol. 174, 147–156. doi: 10.1016/j.jplph.2014.08.021 25462977

[B78] Ratajczak E. Małecka A. Ciereszko I. Staszak A. M. (2019). Mitochondria are important determinants of the aging of seeds. Int. J. Mol. Sci. 20, 1568. doi: 10.3390/ijms20071568 PMC6479606 30925807

[B79] Riewe D. Wiebach J. Altmann T. (2017). Structure annotation and quantification of wheat seed oxidized lipids by high-resolution LC-MS/MS. Plant Physiol. 175, 600–618. doi: 10.1104/pp.17.00470 28801536 PMC5619882

[B80] Rocha J. Nitenberg M. Girard-Egrot A. Jouhet J. Maréchal E. Block M. A.. (2018). Do galactolipid synthases play a key role in the biogenesis of chloroplast membranes of higher plants? Front. Plant Sci. 9. doi: 10.3389/fpls.2018.00126 PMC5809773 29472943

[B81] Rosenzweig C. Elliott J. Deryng D. Ruane A. C. Müller C. Arneth A.. (2014). Assessing agricultural risks of climate change in the 21st century in a global gridded crop model intercomparison. Proc. Natl. Acad. Sci. 111, 3268–3273. doi: 10.1073/pnas.1222463110 24344314 PMC3948251

[B82] Sano N. Rajjou L. North H. M. Debeaujon I. Marion-Poll A. Seo M. (2016). Staying alive: molecular aspects of seed longevity. Plant Cell Physiol. 57, 660–674. doi: 10.1093/pcp/pcv186 26637538

[B83] Saxena K. B. Kumar R.V. Sultana R. (2010). Quality nutrition through pigeonpea–a review. Health 2, 1335–1344. doi: 10.4236/health.2010.211199

[B84] Sew Y. S. Stroher E. Fenske R. Millar A. H. (2016). Loss of mitochondrial malate dehydrogenase activity alters seed metabolism impairing seed maturation and post-germination growth in arabidopsis. Plant Physiol. 171, 849–863. doi: 10.1104/pp.16.01654 27208265 PMC4902577

[B85] Shenzao F. Guangkun Y. Xia X. Shuhua W. Xinghua W. Xinxiong L. (2018). Levels of crotonaldehyde and 4-hydroxy-(E)-2-nonenal and expression of genes encoding carbonyl-scavenging enzyme at critical node during rice seed aging. Rice Sci. 25, 152–160. doi: 10.1016/j.rsci.2018.04.003

[B86] Shuai H. Meng Y. Luo X. Chen F. Zhou W. Dai Y.. (2017). Exogenous auxin represses soybean seed germination through decreasing the gibberellin/abscisic acid (GA/ABA) ratio. Sci. Rep. 7, 1–11. doi: 10.1038/s41598-017-13093-w 28974733 PMC5626727

[B87] Siddiqi E. H. Ashraf M. Al-Qurainy F. Akram N. A. (2011). Salt-induced modulation in inorganic nutrients, antioxidant enzymes, proline content and seed oil composition in safflower (Carthamus tinctorius l.). J. Sci. Food Agric. 91, 2785–2793. doi: 10.1002/jsfa.4522 21717466

[B88] Singh V. Nimbkar N. (2006). 'Safflower (Carthamus tinctorius L.). Genetic Resources, Chromosome Engineering, and Crop Improvement. (Boca Raton, Florida, USA: CRC Press Inc.) 6, 167–194.

[B89] Singh A. K. Varaprasad K. S. Venkateswaran K. (2012). Conservation costs of plant genetic resources for food and agriculture: seed genebanks. Agric. Res. 1, 223–239. doi: 10.1007/s40003-012-0029-3

[B90] Sinha K. Kaur R. Singh N. Kaur S. Rishi V. Bhunia R. K. (2020). Mobilization of storage lipid reserve and expression analysis of lipase and lipoxygenase genes in rice (Oryza sativa var. pusa basmati 1) bran during germination. Phytochemistry 180, 112538. doi: 10.1016/j.phytochem.2020.112538 33091779

[B91] Sultan B. Defrance D. Iizumi T. (2019). Evidence of crop production losses in West Africa due to historical global warming in two crop models. Sci. Rep. 9, 1–15. doi: 10.1038/s41598-019-49167-0 31492929 PMC6731230

[B92] Tan G. Shibasaki R. (2003). Global estimation of crop productivity and the impacts of global warming by GIS and EPIC integration. Ecol. Model. 168, 357–370. doi: 10.1016/S0304-3800(03)00146-7

[B93] Theodoulou F. L. Eastmond P. J. (2012). Seed storage oil catabolism: a story of give and take. Curr. Opin. Plant Biol. 15, 322–328. doi: 10.1016/j.pbi.2012.03.017 22516438

[B94] Tsugawa H. Satoh A. Uchino H. Cajka T. Arita M. Arita M. (2019). Mass spectrometry data repository enhances novel metabolite discoveries with advances in computational metabolomics. Metabolites 9(6), 119. doi: 10.3390/metabo9060119 PMC6630716 31238512

[B95] Ventura L. Donà M. Macovei A. Carbonera D. Buttafava A. Mondoni A.. (2012). Understanding the molecular pathways associated with seed vigor. Plant Physiol. Biochem. 60, 196–206. doi: 10.1016/j.plaphy.2012.07.031 22995217

[B96] Waterworth W. M. Bray C. M. West C. E. (2015). The importance of safeguarding genome integrity in germination and seed longevity. J. Exp. Bot. 66, 3549–3558. doi: 10.1093/jxb/erv080 25750428

[B97] Waterworth W. M. Masnavi G. Bhardwaj R. M. Jiang Q. Bray C. M. West C. E. (2010). A plant DNA ligase is an important determinant of seed longevity. Plant J. 63, 848–860. doi: 10.1111/j.1365-313X.2010.04285.x 20584150

[B98] Wiebach J. Nagel M. Borner A. Altmann T. Riewe D. (2020). Age-dependent loss of seed viability is associated with increased lipid oxidation and hydrolysis. Plant Cell Environ. 43, 303–314. doi: 10.1111/pce.13651 31472094

[B99] Yin G. Whelan J. Wu S. Zhou J. Chen B. Chen X.. (2016). Comprehensive mitochondrial metabolic shift during the critical node of seed ageing in rice. PloS One 11, e0148013. doi: 10.1371/journal.pone.0148013 27124767 PMC4849721

[B100] Yin G. Xin X. Fu S. An M. Wu S. Chen X.. (2017). Proteomic and carbonylation profile analysis at the critical node of seed ageing in oryza sativa. Sci. Rep. 7, 40611. doi: 10.1038/srep40611 28094349 PMC5240128

[B101] Zhang L.-L. Tian K. Tang Z.-H. Chen X.-J. Bian Z.-X. Wang Y.-T.. (2016). Phytochemistry and pharmacology of carthamus tinctorius l. Am. J. Chin. Med. 44, 197–226. doi: 10.1142/S0192415X16500130 27080938

[B102] Zhou W. Chen F. Zhao S. Yang C. Meng Y. Shuai H.. (2019). DA-6 promotes germination and seedling establishment from aged soybean seeds by mediating fatty acid metabolism and glycometabolism. J. Exp. Bot. 70, 101–114, . doi: 10.1093/jxb/ery247 29982626 PMC6305204

[B103] Zhou T. Qiu X. Zhao L. Yang W. Wen F. Wu Q.. (2022). Optimal light intensity and quality increased the saffron daughter corm yield by inhibiting the degradation of reserves in mother corms during the reproductive stage. Ind. Crops Products 176, 114396. doi: 10.1016/j.indcrop.2021.114396

[B104] Zhou W. Yang Y. Zheng C. Luo X. Chandrasekaran U. Yin H.. (2021). Flooding represses soybean seed germination by mediating anaerobic respiration, glycometabolism and phytohormones biosynthesis. Environ. Exp. Bot. 188, 104491. doi: 10.1016/j.envexpbot.2021.104491

[B105] Zinsmeister J. Leprince O. Buitink J. (2020). Molecular and environmental factors regulating seed longevity. Biochem. J. 477, 305–323. doi: 10.1042/BCJ20190165 31967650

