# Supplementary material for: Comparative changes in sugars and lipids show evidence of a critical node for regeneration in safflower seeds during aging
Source: Front Plant Sci. 2022 Oct 27;13:1020478. doi: 10.3389/fpls.2022.1020478 (PMC9661361; doi:10.3389/fpls.2022.1020478)
Supplement: Supplementary file 1 [file DataSheet_1.docx]

Supplementary Material

# Figures S1


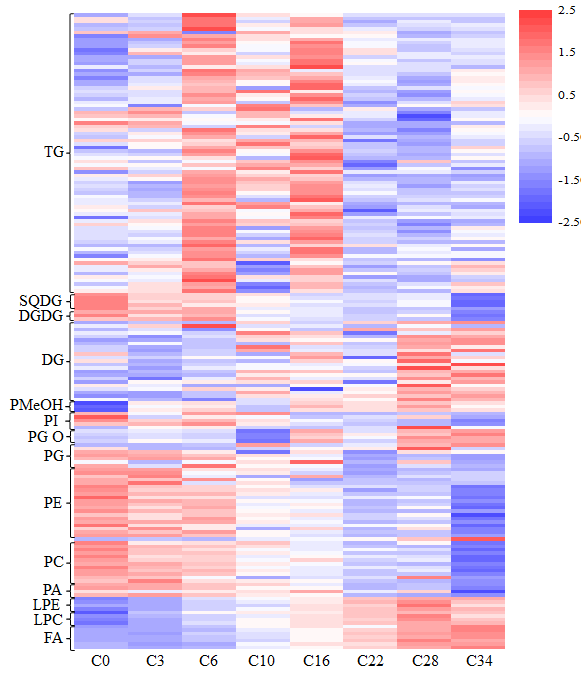


**Supplementary Figure 1.** Effect of CDT process on the lipid of *Carthamus tinctorius* L. seed removed the seedcase. Each coloured bar within a column represents a lipid species in the controlled deterioration treatments. The colour of each bar represents the level of the corresponding lipid species. The data are normalized, with red color indicating high content and blue color indicating low content. Total acyl carbons in ascending order within a class, and total double bonds in ascending order within total acyl carbons.

# Figures S2


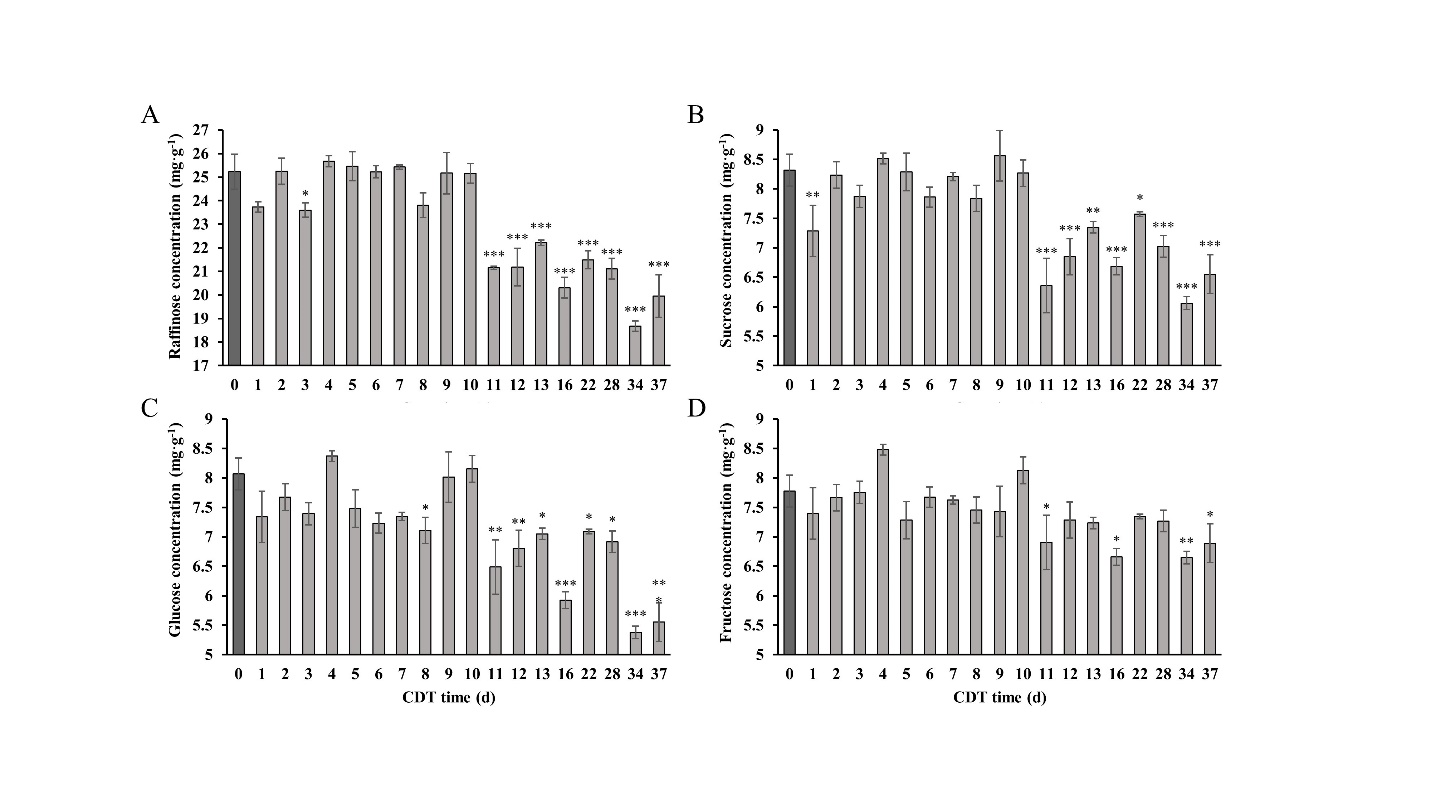


**Supplementary Figure 2.** Changes in the concentration of four sugars with the increase of aging days. (A) Raffinose, (B) Sucrose, (C) Glucose, and (D) Fructose. The values are the mean ± SE (n = 3). The asterisk (*) indicates a significant difference at P<0.05 compared to 0 days of aging (C0).

# Figures S3


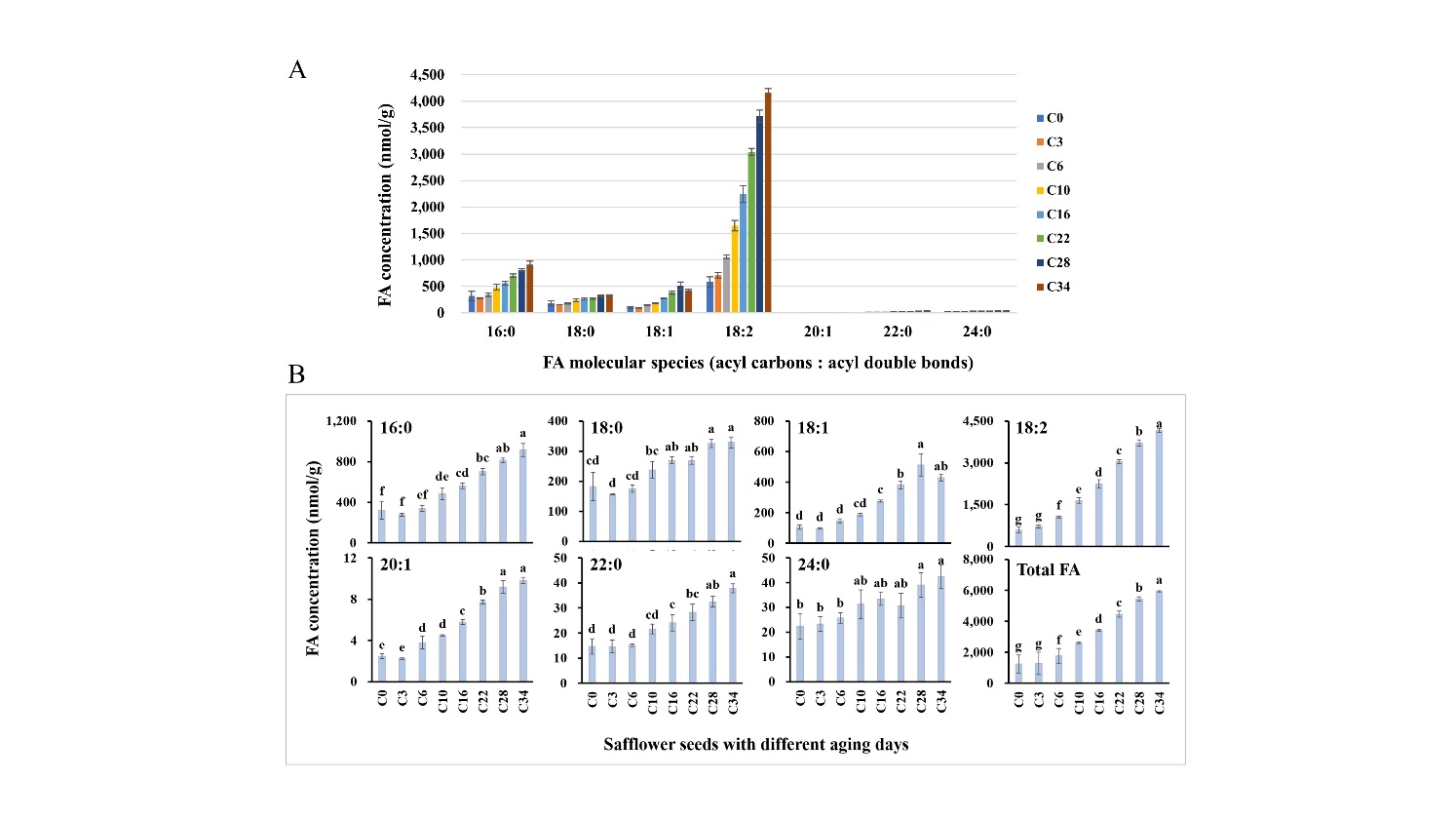


**Supplementary Figure 3.** Changes in molecular species and concentration of FA caused by CDT. (A) Changes in FA levels of different chain lengths. (B) Differential changes of single FA molecules and total. The values are the mean ± SE (n = 3). Different letters in the hatched bars indicates that the value is significantly different (P < 0.05).
